# Chlorinated Guaiane-Type Sesquiterpene Lactones as Cytotoxic Agents against Human Tumor Cells

**DOI:** 10.3390/ijms21249767

**Published:** 2020-12-21

**Authors:** Francisco Estévez-Sarmiento, Ester Saavedra, Mercedes Ruiz-Estévez, Francisco León, José Quintana, Ignacio Brouard, Francisco Estévez

**Affiliations:** 1 Departamento de Bioquímica y Biología Molecular, Instituto Universitario de Investigaciones Biomédicas y Sanitarias (IUIBS), Grupo de Química Orgánica y Bioquímica, Universidad de Las Palmas de Gran Canaria, Unidad Asociada al CSIC, 35016 Las Palmas de Gran Canaria, Spain; francisco.estevez103@alu.ulpgc.es (F.E.-S.); ester.saavedra102@alu.ulpgc.es (E.S.); jose.quintana@ulpgc.es (J.Q.); 2 Recombinetics, Inc., Eagan, MN 55121, USA; mercy.ruiz@recombinetics.com; 3 Department of Drug Discovery and Biomedical Sciences, College of Pharmacy, University of South Carolina, Columbia, SC 29208, USA; jleon@mailbox.sc.edu; 4 Instituto de Productos Naturales y Agrobiología, Consejo Superior de Investigaciones Científicas, 38206 La Laguna, Spain; ibrouard@ipna.csic.es

**Keywords:** apoptosis, cytotoxicity, caspase, poly(ADP-ribose) polymerase, sesquiterpene lactone, guaianolide

## Abstract

Guaiane-type sesquiterpene lactones are naturally occurring compounds which have attracted attention due to their array of biological activities. In this study, chlorinated guaianolides **1**–**8**, isolated from plants of the genus *Centaurea*, were evaluated against the human leukemia cell lines HL-60, U-937, a specific U-937 cell line that overexpresses the anti-apoptotic Bcl-2 protein and the human melanoma cell line SK-MEL-1. This established the relevant structure-growth inhibition relationships. Chlorohyssopifolins A (**1**), C (**3**) and D (**4**) and linichlorin A (**6**) were the most potent compounds in terms of inducing growth inhibition in the four cell lines. IC_50_ values were below 10 μM in all cases. Chlorohyssopifolins A (**1**) and D (**4**) and linichlorin A (**6**) were potent apoptotic inducers in human U-937 leukemia cells, as determined by fluorescent microscopy and flow cytometry, and their mechanism of action was associated with cytochrome *c* release, caspase activation and poly(ADP-ribose)polymerase cleavage. Overall this study shows that guaianolides induce cytotoxicity against human tumor cells and provides important insights into the cell death pathways that are involved.

## 1. Introduction

Natural products may be considered as starting points in the design and development of potential compounds of interest for human medicine. Sesquiterpene lactones are naturally occurring compounds that display a wide array of biological properties and are potential agents against cancer [1,2]. Guaianolides belong to a class of sesquiterpene lactones characterized by three fused rings, two five-membered rings and a seven-membered ring structure with a wide range of biological activities [3]. Some compounds of this class exhibit anti-inflammatory activity due to inhibition of the transcription factor NF-κB [4,5,6]. 

Halogenated compounds generally contain a chlorine atom and about 5000 of them have been isolated from natural sources. They have a wide range of beneficial bioactivities that have led to applications in the pharmaceutical industry. Currently, half of the molecules in high-performance screening contain halogen atoms [6]. Many of these halogenated compounds are chlorohydrins isolated together with their corresponding epoxides [7]. Naturally occurring halogenated sesquiterpene lactones and synthetic derivatives exhibit antitumor and cytotoxic activities and have potential as anticancer agents [8,9].

Apoptosis induction is an important response to many chemotherapeutic agents. This is a mode of regulated cell death characterized by the translocation of phosphatidylserine to the outside of the plasma membrane, formation of apoptotic bodies, chromatin condensation and internucleosomal DNA fragmentation. There are at least two major apoptotic pathways, referred to as the intrinsic pathway and the extrinsic pathway, depending on the cell type [10]. The intrinsic pathway involves the translocation of cytochrome *c* from mitochondria to cytoplasm and caspase-9 activation, which cleaves and activates downstream effector caspases-3, -6 and -7, which in turn trigger the cleavage of key structural and regulatory proteins to effect cell death [11]. The extrinsic pathway involves cell surface death receptors, such as tumor necrosis factor, Fas and TRAIL receptors, and is dependent on the initiator caspase-8, which cleaves and activates the downstream effector caspases [12]. Both caspase-8 and caspase-9 activate caspase-3, which is responsible for breaking specific cellular proteins during apoptosis [13]. Caspase-3 is one of the key executioners of apoptosis, being responsible for the proteolytic cleavage of many key proteins, including the nuclear enzyme poly(ADP-ribose)polymerase which is normally involved in DNA repair [14].

**Figure 1:**
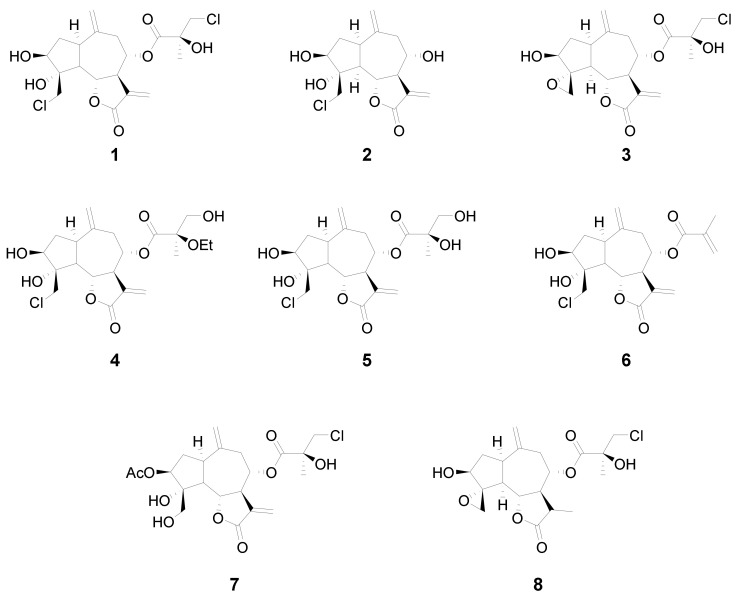
Chemical structures of the guaianolides assayed.

In this study we analyzed the structure–cytotoxicity relationships of eight halogenated guaianolides (seven of them isolated from plants from the genus *Centaurea*, family *Asteraceae*) against human leukemia and melanoma cells. The compounds were selected from our sesquiterpene library, constructed during previous studies that had shown that guaianolides-type sesquiterpene lactones were the most cytotoxic compounds against different cancer cell lines [15]. The guaianolides assayed were isolated from natural sources as previously described [16,17,18]. The chlorinated guaianolides chlorohyssopifolins A (**1**), B (**2**), C (**3**), D (*4*) and E (**5**) were isolated from *Centaurea hyssopifolia* Vahl. Linichlorin A (**6**) and linichlorin C (**7**) were isolated from *Centaurea linifolia* Vahl. The compound 11,13-Dihydrochlorohyssopifolin C (**8**) was obtained from chlorohyssopifolin A through reduction with Zn-Cu followed by epoxide formation with AgNO_3_ [18] (Figure 1). The evaluated guaianolides have the following properties in common: (i) the presence of a double bond as a methylene group at position ten (C-10); (ii) the presence of a hydroxy group and/or an acetyl group in position three of the cyclopentane ring; (iii) different substituents on C-8; and (iv) the presence of a chlorine atom either in position C-15, in the ester moiety or both. Some of the most potent compounds against human tumor cells, i.e., chlorohyssopifolins A (**1**) and D (**4**) and linichlorin A (**6**), were evaluated to determine whether the effects on cell viability were due to the activation of the apoptotic pathway. Specifically, we studied the effects on apoptosis induction and caspase activation. The aim of this study was to explore the structure–cytotoxicity relationships of eight selected sesquiterpene lactones belonging to the guaianolide class against human leukemia and melanoma cells, as well as the mechanism of action of the most potent compounds on apoptosis induction using the human leukemia cells U-937. These cells were chosen since they provided a useful model for the study of neoplasia and therapeutics [19,20]. So far, the potential use of these halogenated guaianolides in antileukemia therapy has been left largely unexplored. We evaluated whether caspase activation and the release of cytochrome *c* were involved in the mechanism of action.

## 2. Results

### 2.1. Chlorinated Guaianolides Inhibit the Growth of Human Tumor Cells

Growth inhibition of human tumor cells in culture was determined by the 3-(4,5-dimethylthiazol-2-yl)-2,5-diphenyl-2*H*-tetrazolium bromide (MTT) dye-reduction assay. A representative microplate is shown in Figure S1. For this assay, cells were incubated with increasing concentrations of guaianolides for 72 h and concentrations inducing a 50% inhibition of cell growth (IC_50_) values were determined by using nonlinear regression (Graph Pad 5.0 software). Guaianolides treatment resulted in a concentration-dependent inhibition of cellular proliferation, except for guaianolides **5** and **8,** which were not cytotoxic, since the IC_50_ values were greater than 30 μM (Supplementary Materials, Figure S2). Most compounds were cytotoxic against all assayed cell lines. In general, no significant differences in cytotoxicity were detected among the four cell lines (Table 1). The most potent compounds against HL-60 cells were chlorohyssopifolins A (**1**), B (**2**), C (**3**) and D (**4**) and linichlorins A (**6**) and C (**7**), with IC_50_ values between 1.2 μM and 7.5 μM, and the less potent compounds were chlorohyssopifolin E (**5**) and 11,13-dihydrochlorohyssopifolin C (**8**). Similar results were obtained for the histiocytic lymphoma U-937 cells. To explore whether the survival protein Bcl-2 is able to protect cells from guaianolides cytotoxicity, the effect against U-937 cell overexpressing Bcl-2 was compared with the parental U-937 cells. As shown in Table 1, the overexpression of Bcl-2 did not protect cells against cytotoxicity. The effects of these guaianolides were also assessed in a melanoma cell line. Melanoma is the most aggressive type of skin cancer and is resistant to most novel therapies. The results revealed that the SK-MEL-1 melanoma cells were also sensitive to the cytotoxic effects of the guaianolides assayed, with chlorohyssopifolin A (**1**) and linichlorin A (**6**) being the most potent compounds, although chlorohyssopifolins C (**3**) and D (**4**) yielded IC_50_ values below 10 μM (Table 1).

The following structure–cytotoxicity relationships were established from the IC_50_ values in the cancer cells assayed: (i) the presence of the α-methylene-γ-lactone functional group is essential for cytotoxicity; (ii) the presence of an ester group on C-8 can both increase (**1** vs. **2**) (at least against U-937, U-937/Bcl-2 and SK-MEL-1) or decrease cytotoxicity (**2** vs. **5**) because it is dependent on the hydrophobicity of the radical; (iii) the introduction of an epoxide functional group on the C-4 of the cyclopentane ring has no effect on cytotoxicity; and (iv) the introduction of an additional alkylating group on the C-8 enhances cytotoxicity (**2** vs. **6**).

To compare the cytotoxic effects of the guaianolides analyzed in this report, we included etoposide as a positive control in all MTT experiments. The IC_50_ values for etoposide were 0.5 ± 0.2 μM, 1.5 ± 0.3 μM and 9.0 ± 3.5 μM in HL-60, U-937 and SK-MEL-1 cells, respectively. 

**Figure 2:**
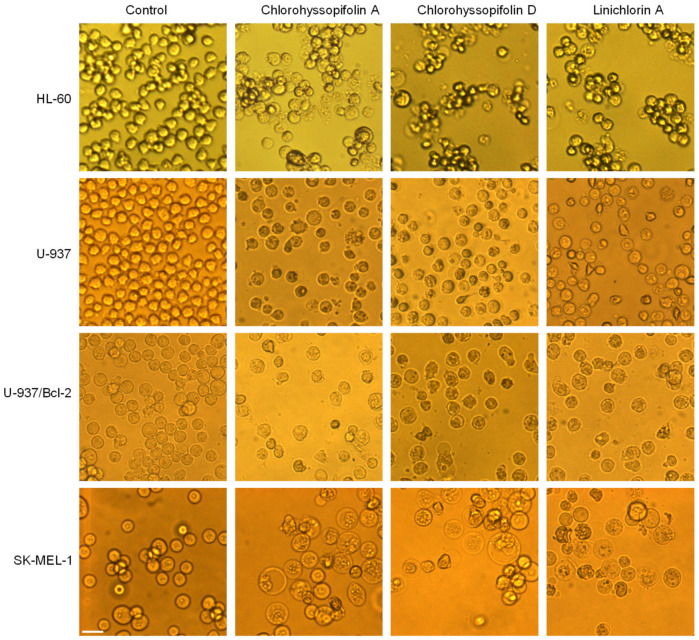
Cells were incubated with dimethylsulfoxide (DMSO) (control) or 10 μM of the compounds shown at the top of the figure for 24 h and images were obtained with an inverted phase-contrast microscope. Original magnification 20×. Bar represents 10 μm.

The visualization of the human tumor cells after treatment with the selected guaianolides chlorohyssopifolins A (**1**) and D (**4**) and linichlorin A (**6**) revealed important morphological changes as well as an important reduction in the number of cells (Figure 2). 

### 2.2. Chlorinated Guaianolides Induce Apoptotic Cell Death

**Figure 3:**
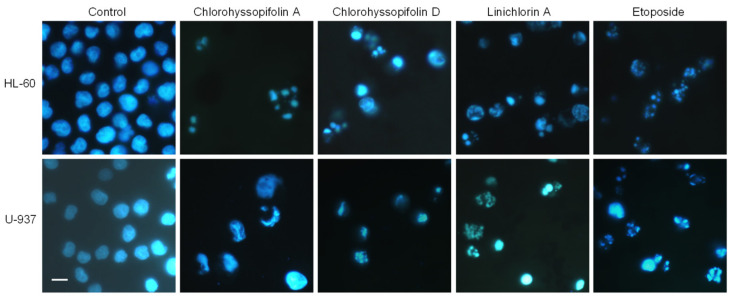
Cells were treated with the indicated guaianolides at a concentration of 10 μM for 24 h, stained with Hoechst 33,258 and visualized under a fluorescence microscope. Etoposide (3 μM) was included as a positive control. Original magnification 40×. Bar represents 10 μm.

In order to determine the mechanism involved in the cytotoxicity induced by chlorohyssopifolins A (**1**) and D (**4**) and linichlorin A (**6**), nuclear morphology was evaluated by fluorescent microscopy after DNA staining with Hoechst 33,258. Cells treated with these guaianolides showed fragmented and condensed chromatin characteristics of apoptotic cell death (Figure 3).

**Figure 4:**
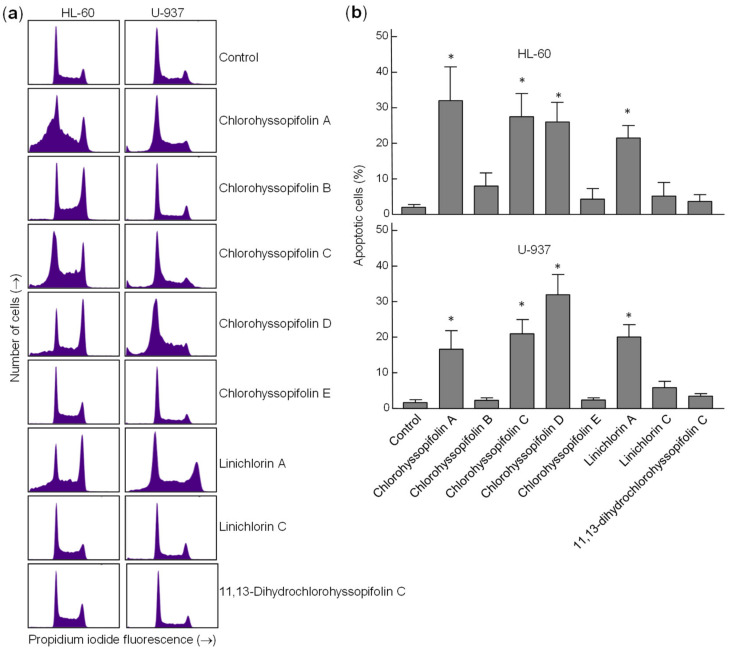
Increase in percentage of apoptotic cells (sub-G_1_ fraction) due to guaianolides. (**a**) Representative histograms of flow cytometry experiments after propidium iodide staining of cells incubated in the presence 10 μM of the indicated guaianolides for 24 h. (**b**) Cells were treated as above and the percentages of apoptotic cells were determined by flow cytometric analysis after propidium iodide staining. Bars represent means ± S.D. of two independent experiments each performed in triplicate. * *p* < 0.05, significantly different from untreated control.

To confirm that the specified guaianolides induce apoptotic cell death, HL-60 and U-937 cells were treated with the selected compounds, analyzed by flow cytometry after propidium iodide staining and the percentage of apoptotic cells (sub-G_1_ fraction) was determined (Figure 4a). The results showed that the percentages of apoptotic cells increased approximately 16-fold (32.0 ± 9.5% vs. 2.0 ± 0.8%), 14-fold (27.5 ± 6.5% vs. 2.0 ± 0.8%), 13-fold (26.0 ± 5.5% vs. 2.0 ± 0.8%) and 13-fold (25.1 ± 3.5% vs. 2.0 ± 0.8%) in HL-60 cells after 24-h exposure of chlorohyssopifolins A (**1**), C (**3**) and D (**4**) and linichlorin A (**6**), respectively. In U-937 cells, the percentages of apoptotic cells increased approximately 10-fold (16.7 ± 5.2% vs. 1.7 ± 0.8%), 12-fold (21.0 ± 4.0% vs. 1.7 ± 0.8%), 19-fold (32 ± 5.7% vs. 1.7 ± 0.8%) and 12-fold (20.1 ± 3.5% vs. 1.7 ± 0.8%) after exposure to chlorohyssopifolins A (**1**), C (**3**) and D (**4**) and linichlorin A (**6**), respectively (Figure 4b). These results reveal that chlorohyssopifolins A (**1**), C (**3**) and D (**4**) and linichlorin A (**6**) were the most potent compounds in inducing apoptosis in U-937 and HL-60 cells. Due to limited availability of naturally occurring compounds, we selected chlorohyssopifolins A (**1**) and D (**4**) and linichlorin A (**6**) for additional experiments. 

### 2.3. Chlorohyssopifolins A *(**1**)* and D *(**4**)* and Linichlorin A *(**6**)* Induce Externalization of Phosphatidylserine, Poly(ADP-ribose)Polymerase (PARP) Cleavage and Cytochrome c Release

**Figure 5:**
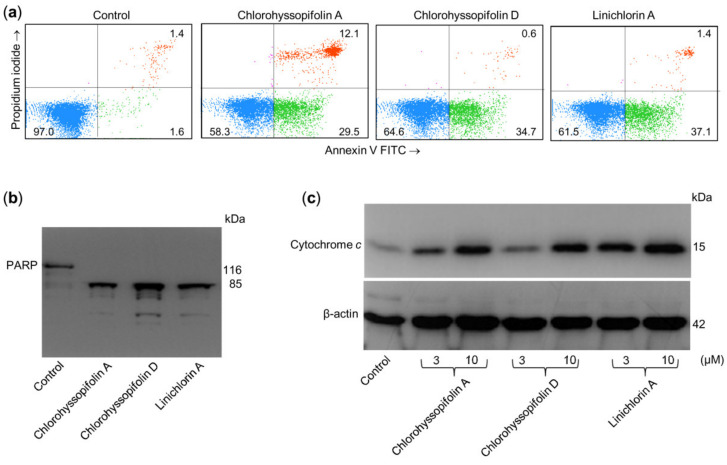
Chlorohyssopifolins A (**1**) and D (**4**) and linichlorin A (**6**) are potent apoptotic inducers in U-937 cells. (**a**) Cells were incubated in the presence of 10 μM of the indicated guaianolides and subjected to flow cytometric analysis after double staining with Annexin V-FITC and propidium iodide. (**b**) Cells were treated as above and Poly(ADP-ribose) polymerase cleavage was determined using Western blot analysis. (**c**) Cytochrome *c* release induced by the specified compounds at the indicated concentrations was analyzed using Western blotting.

Apoptosis induction in U-937 cells was also determined by double staining with annexin V-FITC and propidium iodide and analysis by flow cytometry. As shown in Figure 5a, chlorohyssopifolins A (**1**) and D (**4**) and linichlorin A (**6**) induce phosphatidylserine translocation to the cell surface. A hallmark of apoptosis that indicates caspase activation is cleavage of poly(ADP-ribose)polymerase (PARP), an enzyme involved in DNA repair and a known substrate for caspase-3. To determine whether these guaianolides induce PARP cleavage, U-937 cells were treated with 10 μM of compounds for 24 h and lysates were analyzed by immunoblotting. The results revealed that the guaianolides induced the hydrolysis of the 116 kDa full-length PARP protein to yield the 85 kDa fragment (Figure 5b). 

The mitochondrial apoptotic pathway involves the release of cytochrome *c* from mitochondria. To investigate whether guaianolides promote cytochrome *c* release, cytosolic fractions were analyzed using Western blotting. As shown in Figure 5c, guaianolides treatment resulted in a concentration-dependent release of the mitochondrial protein cytochrome *c* into the cytosol.

### 2.4. Bcl-2 Over-Expression Did Not Block Apoptosis Induction by Chlorohyssopifolins A *(**1**)* or D *(**4**)* or Linichlorin A *(**6**)*

**Figure 6:**
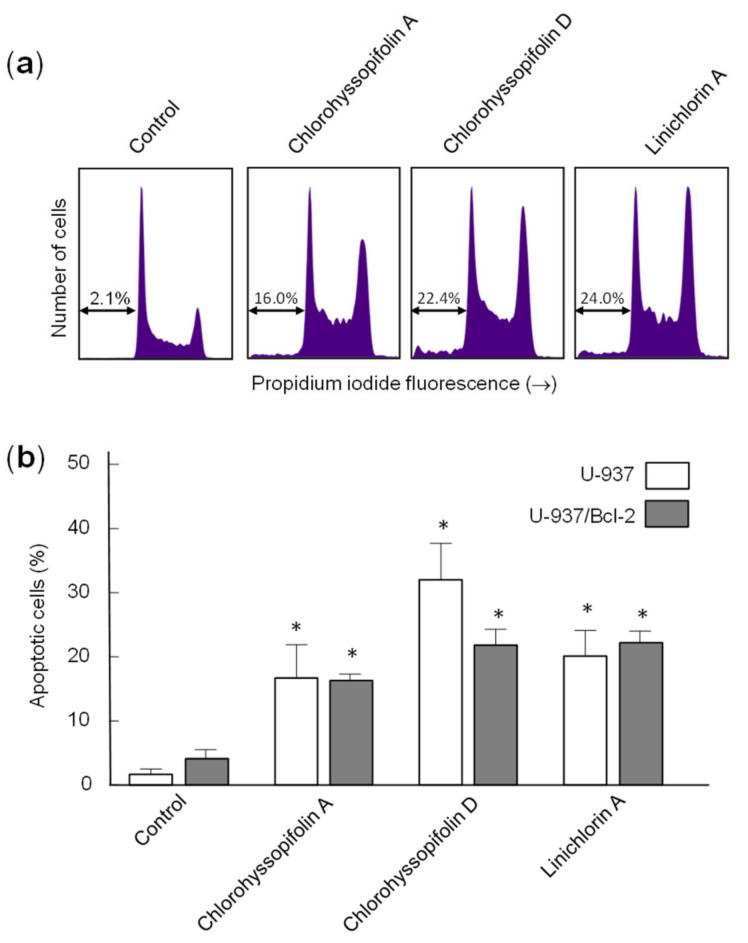
(**a**) Representative histograms obtained by flow cytometry after staining the U-937/Bcl-2 cells with propidium iodide. (**b**) Cells were incubated in the absence or presence of the indicated guaianolides at 10 μM concentration for 24 h and the percentages of apoptotic cells were determined by flow cytometric analysis after propidium iodide staining. Bars represent means ± S.D. of two independent experiments each performed in triplicate. * *p* < 0.05, significantly different from untreated control.

Human B-cell lymphoma-2 (Bcl-2) is a key protein involved in apoptosis inhibition through its regulation of mitochondrial membrane potential and cytochrome *c* release needed for the activation of caspase-9. Therefore, it should be interesting to clarify whether this protein is able to protect cells against the increase of apoptotic cells triggered by chlorohyssopifolins A (**1**) and D (**4**) and linichlorin A (**6**). To this end, we tested the cell line overexpressing Bcl-2 (U-937/Bcl-2) and observed that the overexpression of this survival factor did not block apoptosis triggered by chlorohyssopifolin A (**1**) or linichlorin A (**6**), but weakly decreased the percentage of apoptotic cells induced by chlorohyssopifolin D (**4**) (Figure 6a,b). As shown in Figure 6, the percentages of apoptotic cells increased approximately 8-fold (16.3 ± 1.0% vs. 2.0 ± 0.8%), 11-fold (21.8 ± 2.5% vs. 2.0 ± 0.8%) and 11-fold (22.2 ± 1.8% vs. 2.0 ± 0.8%) after 24 h of treatment with chlorohyssopifolins A (**1**) and D (**4**) and linichlorin A (**6**), respectively. These increases in the percentages of apoptotic cells were similar to those obtained in the parental U-937 cells indicated above except for those of chlorohyssopifolin D (**4**). For this compound the percentages of apoptotic cells increased to 30% and 22% for U-937 and U-937/Bcl-2, respectively. It is important to note that the flow cytometry experiments were carried out after 24 h of treatment. However, as indicated above, these guaianolides were able to induce cell death and showed similar IC_50_ values in both cell lines, i.e., the U-937 cell line overexpressing human Bcl-2 protein (U-937/Bcl-2) and the parental U-937 cell line, as determined by the MTT assay which was performed for 72 h (Table 1). These results indicate that, in addition to apoptosis, other mechanisms that induce cell growth inhibition in U-937/Bcl-2, such as cell cycle arrest, may be involved by these guaianolides.

### 2.5. Chlorohyssopifolins A *(**1**)* and D *(**4**)* and Linichlorin A *(**6**)* Stimulate Caspase Activity and Processing

**Figure 7:**
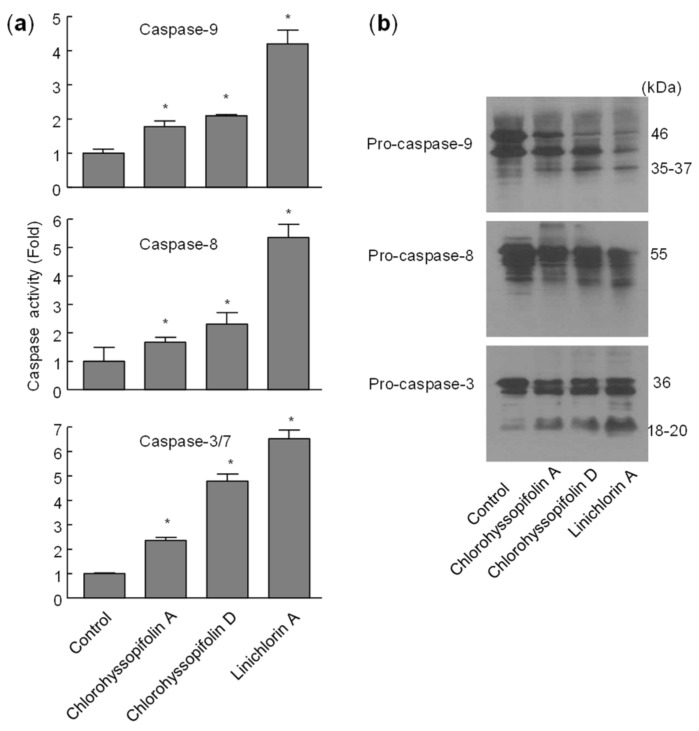
Chlorohyssopifolins A (**1**) and D (**4**) and linichlorin A (**6**) induce activation and processing of caspases in U-937 cells. (**a**) Cells were treated with 10 μM of the indicated guaianolide, harvested at 24 h and cell lysates were assayed for caspase-9, caspase-8 and caspase-3/7 activities using colorimetric substrates. Results are expressed as factorial increases in caspase activity compared with the control. Values represent means ± S.D. of two independent experiments each performed in triplicate. * *p* < 0.05, significantly different from untreated control. (**b**) Processing of caspases in response to chlorohyssopifolins A (**1**) and D (**4**) and linichlorin A (**6**). Cells were incubated with 10 μM of the indicated guaianolide for 24 h and the corresponding caspases were determined by Western blot analysis.

To define which caspases were involved during apoptosis induced by the selected guaianolides, the enzymatic activity of lysates on tetrapeptide substrates LEHD-*p*NA (for caspase-9), IETD-*p*NA (for caspase-8) and DEVD-*p*NA (for caspase-3/7) was analyzed after 24-h exposure to chlorohyssopifolins A (**1**) and D (**4**) and linichlorin A (**6**). As shown in Figure 7a, there was an approximately twofold increase in caspase-9 activity in response to chlorohyssopifolins A (**1**) and D (**4**) and a fourfold increase after treatment with linichlorin A (**6**). A similar activation of caspase-8 was also observed. The executioner caspase-3/7 activity increased approximately twofold, fivefold and sevenfold after treatment with chlorohyssopifolins A (**1**) and D (**4**) and linichlorin A (**6**), respectively. Processing of initiator caspases, caspase-8 and -9, as well as the main executioner caspase, caspase-3, was also investigated. To this end, cells were treated with chlorohyssopifolins A (**1**) and D (**4**) and linichlorin A (**6**) for 24 h and lysates were analyzed using Western blotting. The results showed processing of pro-caspase-3 by the three guaianolides in U-937 cells (Figure 7b). Processing of caspase-8 and -9 was also detected, linichlorin A (**6**) being the most potent compound in inducing procaspase processing, in accordance with enzymatic activities of caspase-3-like proteases (caspase-3/7) and of caspase-8 and -9.

## 3. Discussion 

In recent years there has been a renewed interest in naturally occurring compounds as potential chemopreventive and chemotherapeutic treatments against cancer. It is estimated that about 60% of compounds that are used in the fight against cancer may be considered as derived from natural products. Among these compounds, sesquiterpene lactones have received considerable attention in the last 20 years [21]. Sesquiterpenes are compounds of fifteen carbon atoms with different skeletons, mainly (but not exclusively) germacrane, guaiano and eudesmane skeletons. Sesquiterpene lactones are characterized by an α-methylene-γ-lactone functional group which acts as an electrophile group able to react with specific nucleophiles functional groups in Michael-type addition reaction [4]. The germacranolide parthenolide is one of the most studied sesquiterpene lactones. It exhibits anti-inflammatory activity and promising potential anticancer activity [22,23]. The guaianolide-type sesquiterpene lactones have also attracted attention because, in general, they exhibit a higher cytotoxic activity compared with the other types of sesquiterpene lactones [15]. For example, arglabin and micheliolide can selectively inhibit acute myelogenous leukemia stem or progenitor cell growth and dehydroleucodine displayed antitumour activity in a preclinical melanoma model [24,25]. Interestingly, phase II clinical trials were recently completed [3] for mipsagargin, an analogue of the potent sarco-endoplasmic reticulum Ca^2+^-ATPase inhibitor thapsigargin in several types of cancer.

Here we explored the structure–cytotoxicity relationships of eight chlorinated guaianolides, seven obtained from natural sources and one as a semisynthetic derivative, using human leukemia and melanoma cell lines as models. These compounds were characterized by the presence of exocyclic double bonds on carbons C-10 and C-11 and different substituents on carbons C-3, C-4 and C-8. The results of the studies of structure–cytotoxicity relationships revealed that: (i) the reduction of the double bond 11,13 blocked inhibitory activity in tumor cells, independent of the presence of an additional reactive electrophilic group; (ii) acetylation of hydroxy group on C-3 did not enhance cytotoxicity in comparison with the corresponding alcohol; (iii) the introduction of an ester group on C-8 amplified or decreased cytotoxicity; (iv) the increase in polarity of the hydrocarbon chain of the ester group on C-8 reduced cytotoxicity; (v) the introduction of an alkylating group such as an epoxide on C-4 did not enhance cytotoxicity but the introduction of an enone on C-8 did. 

The importance of the α-methylene-γ-lactone in determining cytotoxicity in the presence of additional reactive electrophilic groups has been described for repin, a guaianolide isolated from *Centaurea repens* [26]. Previous studies have shown that chlorohyssopifolin A (**1**) inhibits cell viability of several cancer cell lines, including 1A9 (ovarian cancer), KB (nasopharyngeal cancer) and KB-V (vincristine-resistant KB subline), and that chlorohyssopifolin C (**3**) showed significant cytotoxicity against MCF-7 (estrogen receptor positive breast cancer). In that study the chlorohyssopifolins A (**1**) and C (**3**) were not assayed against human melanoma cells and the mechanism of cell death was not explored [8]. Similar to our results in human leukemia and melanoma cells, the study showed that the chlorohydrin on C-4 may be modified to an epoxide on the cyclic skeleton with no change in cytotoxicity, as demonstrated by the comparison of the IC_50_ values of chlorohyssopifolin A (**1**) and chlorohyssopifolin C (**3**). In addition, the presence of a diol in the side chain on C-8 (chlorohyssopifolin E (**5**)) rather than a chlorohydrin (chlorohyssopifolin A (**1**)) abolished activity. A similar observation has been described for the comparison between babylin A, which contains a diol in the side chain, and chlorohyssopifolin C (**3**) which contains a chlorohydrin in the side chain [8].

Interestingly, the guaianolides were able to inhibit cell viability in U-937/Bcl-2, a subline that overexpresses the survival protein Bcl-2, which has been involved in chemoresistance, especially in hematologic malignancies [27]. These results suggest that guaianolides seem capable of blocking the growth of human tumor cells by inactivation of the mitochondrial protection by Bcl-2. Moreover, SK-MEL-1 melanoma cells were sensitive to chlorohyssopifolins A (**1**), C (**3**) and D (**4**) and linichlorin A (**6**), emphasizing the potential of these compounds, given that melanoma is the most aggressive and lethal skin cancer. 

The results of this work revealed that linichlorin A (**6**) was one of the most potent guaianolides in reducing the cell viability of the four human tumor cells assayed. Linichlorin A (**6**) has been reported as a negative regulator of the degradation of the cyclin-dependent kinases inhibitor p27^Kip1^. This guaianolide inhibits the *in vitro* ubiquitination of p27^Kip1^ by SCF^Skp2^—the ubiquitin ligase (SCF) complex with S-phase kinase-associated protein 2- stabilizes p27^Kip1^ levels in HeLa cells and inhibits the growth of human and mouse cancer cells [28]. Linichlorin A (**6**) has been shown to display substantial, selective antiproliferative activity against cancer and transformed cells in the micromolar range. The IC_50_ values reported for linichlorin A (**6**) in HeLa (human cervix carcinoma), tsFT210 (mouse tumor cells) and NIH3T3 (mouse immortalized cells) were 3.2, 1.6 and 12.7 μM, respectively, as determined by WST-8 assay for 48 h. However, little is known about the mechanism of cell death induced by linichlorin A (**6**).

Flow cytometry experiments in the present study revealed that inhibition of cell viability by chlorohyssopifolins A (**1**), C (**3**) and D (**4**) and linichlorin A (**6**) was accompanied by an increase in the sub-G_1_ fraction. The selected guaianolides chlorohyssopifolins A (**1**) and D (**4**) and linichlorin A (**6**) were able to induce nuclear morphological changes, such as fragmentation and condensation of chromatin, characteristic of apoptotic cell death. Experiments using U-937 cells as a model confirmed that these compounds are potent apoptotic inducers, as demonstrated by phosphatidylserine externalization and poly(ADP-ribose)polymerase cleavage. In addition, selected guaianolides induced a concentration-dependent release of mitochondrial apoptogenic cytochrome *c*, indicating that the intrinsic apoptotic pathway may play a key role in cell death. Enzymatic analysis revealed activation of caspase-9 and caspase-3, in accordance with the release of cytochrome *c*. Activation of caspase-8 was also detected in extracts of selected guaianolides-treated U-937 cells. In order to identify the primary targets and early mechanism of action of selected sesquiterpene lactones on U-937 cells, we used concentrations close to or threefold higher than the antiproliferative IC_50_ values, which were determined at 72 h of treatment, while flow cytometry and assays of caspase activity were analyzed after 24 h of treatment. Concentrations close to the IC_50_ values of selected sesquiterpene lactones were sufficient to trigger apoptosis. It is interesting to note that a low concentration (3 μM) of chlorohyssopifolins A (**1**) and D (**4**) and linichlorin A (**6**) was sufficient to induce cytochrome *c* release in U-937 cells. Taken together, these results indicate that both the extrinsic and the intrinsic pathways play an important role in cell death induced by chlorohyssopifolins A (**1**) and D (**4**) and linichlorin A (**6**). 

In conclusion, chlorohyssopifolins A (**1**) and D (**4**) and linichlorin A (**6**) were shown to induce apoptosis via caspase activation. Although more research must be carried out to uncover the detailed pathway of cell death, these compounds are potentially interesting and should be considered for further preclinical and *in vivo* testing.

## 4. Materials and Methods

### 4.1. Drugs and Reagents

The guaianolides assayed were isolated from natural sources as previously described. Five sesquiterpene lactones containing chlorine, chlorohyssopifolins A (**1**), B (**2**), C (**3**), D (**4**) and E (**5**) were isolated from *Centaurea hyssopifolia* Vahl, which is an endemic dominant species on Iberian gypsum soils in central Spain [15,16]. Linichlorin A (**6**) is a sesquiterpene lactone that was first isolated from *Centaurea linifolia* Vahl, a native plant from the eastern part of Spain and Italy [17]. The chemical structures of sesquiterpene lactones were determined spectroscopically (proton nuclear magnetic resonance, infrared spectroscopy and mass spectrometry) as described previously. ^1^H- NMR, ^13^C-NMR and mass spectra of guaianolides are shown in Figures S3–S33 in Supplementary Materials.

### 4.2. Cell Culture

The human acute myeloid leukemia HL-60 (DSMZ N° ACC 3), the human histiocytic lymphoma U-937 (DSMZ N° ACC 5) and the human melanoma SK-MEL-1 (DSMZ N° ACC 303) were obtained from the German Collection of Microorganisms and Cell Cultures (Braunschweig, Germany), cultured in suspension in RPMI 1640 medium containing 10% (*v*/*v*) fetal bovine serum and maintained at 0.5–1.0 × 10^6^ cells/mL, except SK-MEL-1 cells, which were maintained at 0.1–0.3 × 10^6^ cells/mL. The U-937 cell line overexpressing human Bcl-2 (designated U-937/Bcl-2) was donated by Dr. Jacqueline Bréard (Faculté de Pharmacie Paris-Sud, Chatenay-Malabry, France) and cultured in RPMI 1640 medium containing 10% (*v*/*v*) fetal bovine serum at 37 °C in a humidified atmosphere containing 5% CO_2_. Cell viability was determined by the trypan blue exclusion test. Cells were resuspended in fresh medium 24 h before treatments to ensure the exponential growth. HL-60 and U-937 cells exhibited characteristic doubling times of about 25 h and 35 h, respectively, while SK-MEL-1 cells exhibited a doubling time of several days (about 48–72 h). Stock solutions of 50 mM guaianolides were made in dimethylsulfoxide (DMSO) and aliquots were frozen at −20 °C. Further dilutions were made in culture medium immediately prior to use. In all experiments, the final concentration of DMSO did not exceed 0.2% (*v*/*v*), a concentration that was nontoxic to the cells.

### 4.3. Assay for Growth Inhibition

The effects of guaianolides on the cell viability of human tumor cells were assessed using the colorimetric 3-(4,5-dimethyl-2-thiazolyl)-2,5-diphenyl-2*H*-tetrazolium bromide (MTT) assay [29]. Exponentially growing cells (4000 for HL-60 and U-937; 6000 for SK-MEL-1) were seeded in 96-well microculture plates with increasing concentrations of guaianolides for 72 h at 37 °C. Surviving cells were detected based on their ability to metabolize MTT in formazan crystals, measuring the absorbance at 570 nm, and the IC_50_ (concentrations inducing a 50% inhibition of cell growth) values were calculated graphically using the curve-fitting algorithm of the computer software Prism 5.0 (GraphPad, La Jolla, CA, USA). Values were calculated as means ± SD from three to five independent experiments, each performed in triplicate.

### 4.4. Fluorescent Microscopy

Cells were treated with the corresponding compound for the specified time period and then fixed with 3% paraformaldehyde for 10 min at room temperature. Cells were then stained with bisbenzimide (Hoechst 33258) for 15 min and visualized under a fluorescence microscope.

### 4.5. Analysis by Flow Cytometry

After treatment with guaianolides, cells were fixed in 75% ethanol at −20 °C for at least 1 h, stained with propidium iodide and analyzed by flow cytometry using a BD FACS Verse cytometer. Apoptosis was quantified using an Annexin V-FITC apoptosis detection kit (BD Pharmingen, San Diego, CA, USA), performed according to the manufacturer’s protocol.

### 4.6. Caspase Activity

Caspase activity was determined in cell lysates using specific colorimetric substrates for caspase-3/7, caspase-8 and caspase-9 [30]. Briefly, after treatments cells were washed twice in phosphate buffer saline, they were resuspended in 50 mM HEPES pH 7.4, 0.1 mM EDTA, 1 mM dithiothreitol, 0.1% Chaps, and lysed by pushing them several times through a 22-gauge needle. The cell lysates were centrifuged at 17,000× *g* at 4 °C for 10 min and the supernatants were analyzed for protein concentration and for caspase activity. Protein concentration was determined by the Bradford assay and caspase activity by the net increase of absorbance at 405 nm. The specific substrates for caspase-3/7, -8 and -9 were DEVD-*p*NA, IETD-*p*NA and LEHD-*p*NA, respectively.

### 4.7. Western Blot

Immunoblot analyses of caspases and PARP were performed as previously described [31]. Briefly, cells were treated with the specified compounds, washed twice with PBS and then lysed in a buffer containing 50 mM Tris-HCl, pH 8.0, 150 mM sodium chloride, 1.0% Triton X-100, 0.1 mM phenylmethylsulfonylfluoride and leupeptin, pepstatin A and aprotinin (1 μg/mL each). Equal amounts of proteins were denatured in 2x Laemli buffer (0.125 M Tris-HCl pH 6.8, 4% SDS, 10% mercaptoethanol, 20% glycerol and 0.004% bromophenol blue) at 95 °C for 5 min. The samples were separated on a 7.5% (PARP) or 12.5% (caspases) SDS-polyacrylamide gel and electrotransferred to a polyvinylidene difluoride (PVDF) membrane. After blocking the membrane with 5% nonfat milk in Tris-buffered saline containing 0.1% Tween-20 for 1 h, it was incubated with the corresponding primary antibodies followed by the corresponding secondary antibodies. The antigen–antibodies complexes were visualized by enhanced chemiluminiscence.

For the cytosolic fractions, cells were washed twice with PBS, resuspended in 20 mM Hepes-KOH pH 7.5, 10 mM KCl, 1.5 mM MgCl_2_, 1 mM EDTA, 1 mM EGTA, 0.1 mM phenylmethylsulfonylfluoride, 1 mM dithiothreitol, 250 mM sucrose and 1 μg/mL leupeptin, pepstatin A and aprotinin, and lysed by pushing them several times through a 22-gauge needle. Lysates were centrifuged at 1000× *g* for 5 min at 4 °C and the supernatants were spun down again at 105,000× *g* for 45 min at 4 °C. The resulting supernatants were used as the soluble cytosolic fraction.

The primary antibodies used for Western blots were purchased from the following companies: anti-caspase-3 (ADI-AAP-113), -8 (ADI-AAM-118) and -9 (ADI-AAM-139) from Enzo (Plymouth Meeting, PA, USA); anti-poly(ADP-ribose) polymerase (PARP) (551024) and anti-cytochrome *c* (556433) from BD Pharmingen (San Diego, CA, USA); anti-β-actin (clone AC-74, A2228) from Sigma-Aldrich (Saint Louis, MO, USA). Secondary antibodies (NA9310 and NA9340) were from GE Healthcare (Little Chalfont, UK). PVDF membranes were from Millipore (Temecula, CA, USA).

### 4.8. Statistical Analysis

Statistical differences between means of control and treated samples were tested using Student’s *t*-test. *p*-values below 0.05 were considered as statistically significant.

## Figures and Tables

**Table 1:** Effects of guaianolides on cell viability of human tumor cell lines.

	IC_50_ (µM)			
	HL-60	U-937	U-937/Bcl-2	SK-MEL-1
Chlorohyssopifolin A (**1**)	5.9 ± 0.9	2.9 ± 1.2	1.7 ± 1.5	3.4 ± 0.6
Chlorohyssopifolin B (**2**)	7.5 ± 1.5	9.2 ± 2.7	13.4 ± 3.7	11.5 ± 2.0
Chlorohyssopifolin C (**3**)	4.1 ± 2.1	5.2 ± 2.5	1.2 ± 0.8	6.9 ± 0.9
Chlorohyssopifolin D (**4**)	4.9 ± 1.8	3.9 ± 1.4	1.0 ± 0.6	7.6 ± 1.4
Chlorohyssopifolin E (**5**)	–	–	–	–
Linichlorin A (**6**)	1.2 ± 0.6	1.9 ± 0.5	2.9 ± 1.8	3.6 ± 1.3
Linichlorin C (**7**)	4.9 ± 1.7	5.0 ± 0.4	12.8 ± 1.9	10.5 ± 0.4
11,13-Dihydrochlorohyssopifolin C (**8**)	–	–	–	–

The IC_50_ values were calculated from cells treated for 72 h using the methodology described in the Materials and Methods section. The data shown represent the mean ± SD of 3–5 independent experiments with three determinations each. – means not active, IC_50_ values > 30 μM. HL-60, U-937, U-937/Bcl-2: acute myeloid leukemia; SK-MEL-1: melanoma.

## Supplementary Materials

Supplementary materials can be found at https://www.mdpi.com/1422-0067/21/24/9767/s1. Representative MTT experiment of HL-60 cells treated with increasing concentrations of guaianolides for 72 h (Figure S1), dose-response curves of guaianolides **1**–**8** on human tumor cells viability (Figure S2) and ^1^H-NMR and ^13^C-NMR spectra, as well as high and low mass spectra (Figures S3–S33).
