# Supplementary material for: Chlorinated Guaiane-Type Sesquiterpene Lactones as Cytotoxic Agents against Human Tumor Cells
Source: Int J Mol Sci. 2020 Dec 21;21(24):9767. doi: 10.3390/ijms21249767 (PMC7767465; doi:10.3390/ijms21249767)
Supplement: Supplementary file 1 [file ijms-21-09767-s001.pdf]

Supplementary Materials

Chlorinated guaiane-type sesquiterpene lactones as cytotoxic agents against human tumor cells

Francisco Estévez-Sarmiento[1], Ester Saavedra[1], Mercedes Ruiz-Estévez[2], Francisco León[3], José Quintana[1], Ignacio Brouard[4], and Francisco Estévez[1,*]

[1] Departamento de Bioquímica y Biología Molecular, Instituto Universitario de Investigaciones Biomédicas y Sanitarias (IUIBS), Grupo de Química Orgánica y Bioquímica, Universidad de Las Palmas de Gran Canaria, Unidad Asociada al CSIC, 35016 Las Palmas de Gran Canaria, Spain; francisco.estevez103@alu.ulpgc.es (F.E.-S.) ; ester.saavedra102@alu.ulpgc.es (E.S.) ; jose.quintana@ulpgc.es (J.Q.)
[2] Recombinetics, Inc., Eagan, MN 55121, USA; mercy.ruiz@recombinetics.com (M.R.-E.)
[3] Department of Drug Discovery and Biomedical Sciences. College of Pharmacy. University of South Carolina, Columbia, SC 29208, USA; jleon@mailbox.sc.edu (F.L.)
[4] Instituto de Productos Naturales y Agrobiología, Consejo Superior de Investigaciones Científicas, 38206 La Laguna, Spain; ibrouard@ipna.csic.es (I.B.)
* Correspondence: francisco.estevez@ulpgc.es; Tel.: (+34 928 451443)

Table of Contents

Figure S1: Representative MTT experiment of HL-60 cells treated with increasing concentrations of guaianolides for 72 h.
Figure S2: Dose-response curves of guaianolides **1-8** on human tumor cells viability.
Figure S3: ^{1}H-NMR (500 MHz, Acetone-d6) Spectrum of Compound **1**
Figure S4: ^{13}C-NMR (125 MHz, Acetone-d6) Spectrum of Compound **1**
Figure S5: HRESI-MS Spectrum of Compound **1**
Figure S6: ESI-MS Spectrum of Compound **1**
Figure S7: ^{1}H -NMR (500 MHz, DMSO-d6) Spectrum of Compound **2**
Figure S8: HRESI-MS Spectrum of Compound **2**
Figure S9: ESI-MS Spectrum of Compound **2**
Figure S10: ^{1}H -NMR (500 MHz, Acetone-d6) Spectrum of Compound **3**
Figure S11: ^{13}C -NMR (125 MHz, Acetone-d6) Spectrum of Compound **3**
Figure S12: HRESI-MS Spectrum of Compound **3**
Figure S13: ESI-MS Spectrum of Compound **3**
Figure S14: ^{1}H -NMR (500 MHz, Acetone-d6) Spectrum of Compound **4**
Figure S15: ^{13}C -NMR (125 MHz, Acetone-d6) Spectrum of Compound **4**
Figure S16: HRESI-MS Spectrum of Compound **4**
Figure S17: ESI-MS Spectrum of Compound **4**
Figure S18: ^{1}H -NMR (500 MHz, Acetone-d6) Spectrum of Compound **5**
Figure S19: ^{13}C -NMR (125 MHz, Acetone-d6) Spectrum of Compound **5**
Figure S20: HRESI-MS Spectrum of Compound **5**
Figure S21: ESI-MS Spectrum of Compound **5**
Figure S22: ^{1}H -NMR (500 MHz, $CDCl_3$) Spectrum of Compound **6**
Figure S23: ^{13}C -NMR (125 MHz, $CDCl_3$) Spectrum of Compound **6**
Figure S24: HRESI-MS Spectrum of Compound **6**
Figure S25: ESI-MS Spectrum of Compound **6**

Figure S26: ^{1}H -NMR (500 MHz, Acetone-d6) Spectrum of Compound **7**
Figure S27: ^{13}C -NMR (125 MHz, Acetone-d6) Spectrum of Compound **7**
Figure S28: HRESI-MS Spectrum of Compound **7**
Figure S29: ESI-MS Spectrum of Compound **7**
Figure S30: ^{1}H -NMR (500 MHz, Acetone-d6) Spectrum of Compound **8**
Figure S31: ^{13}C -NMR (125 MHz, Acetone-d6) Spectrum of Compound **8**
Figure S32: HRESI-MS Spectrum of Compound **8**
Figure S33: ESI-MS Spectrum of Compound **8**

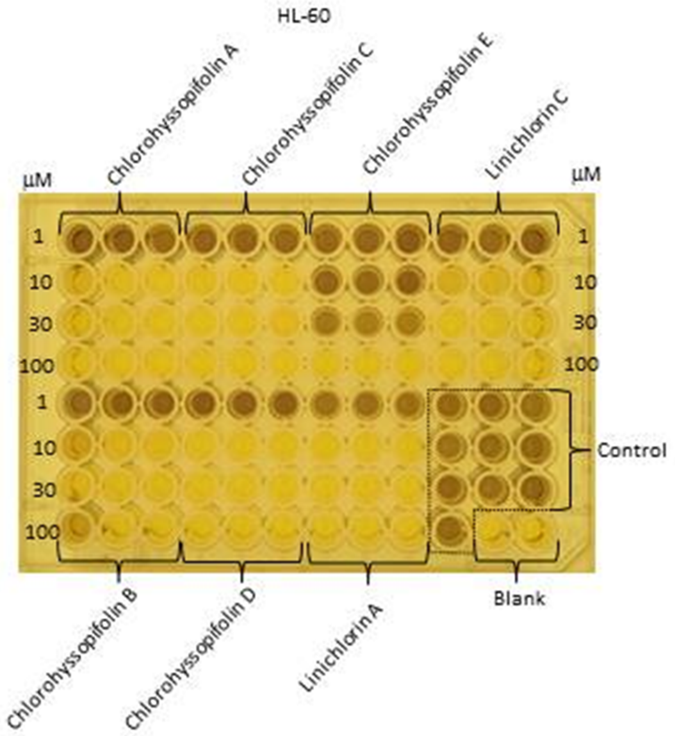

Figure S1. Representative MTT experiment of HL-60 cells treated with increasing concentrations of guaianolides for 72 h.

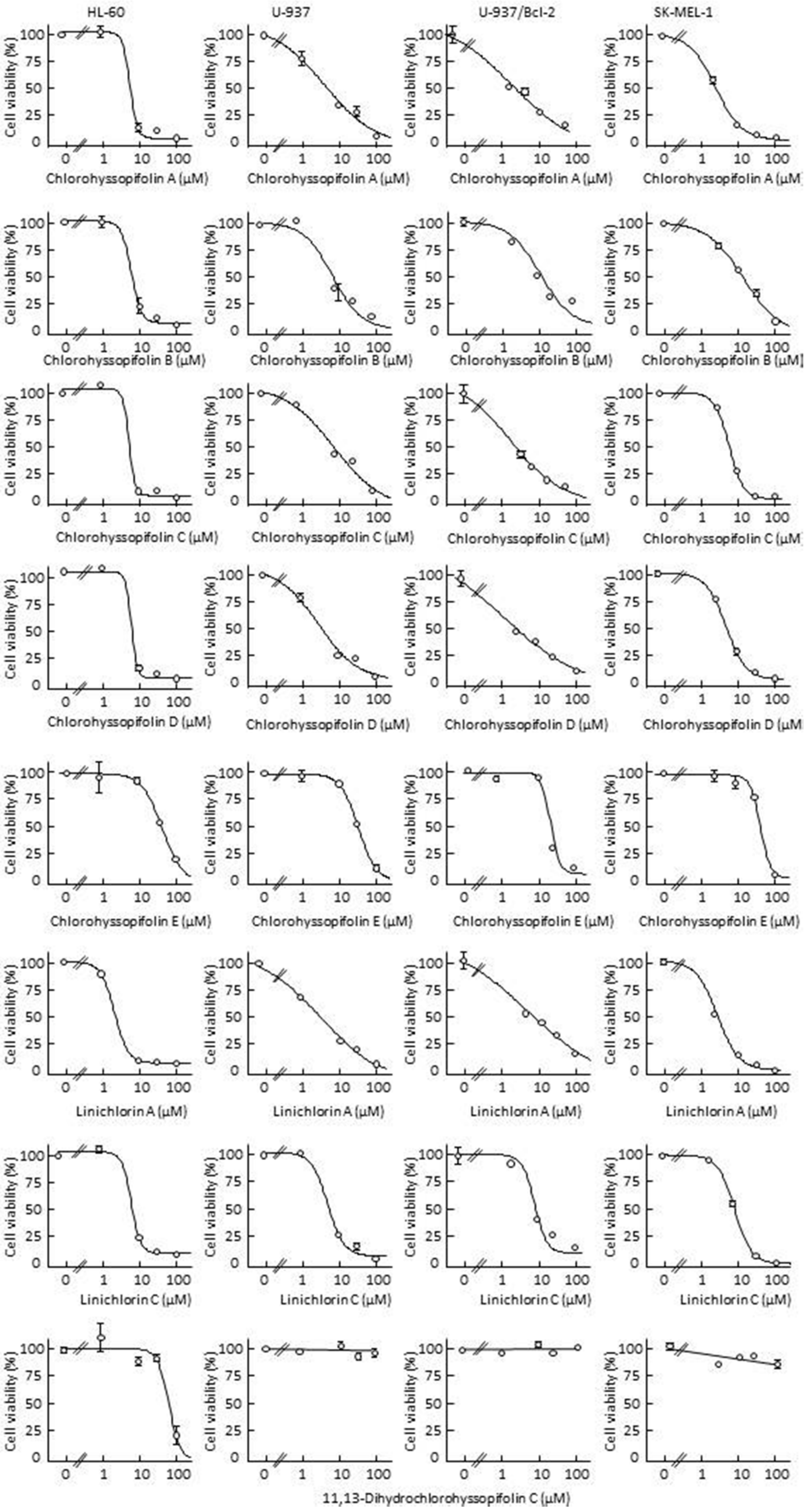

Figure S2. Dose-response curves of guaianolides **1-8** on human tumor cells viability. Cells were incubated in the presence of increasing concentrations of the indicated guaianolide for 72 h, and thereafter cell viability was determined by the MTT assay.

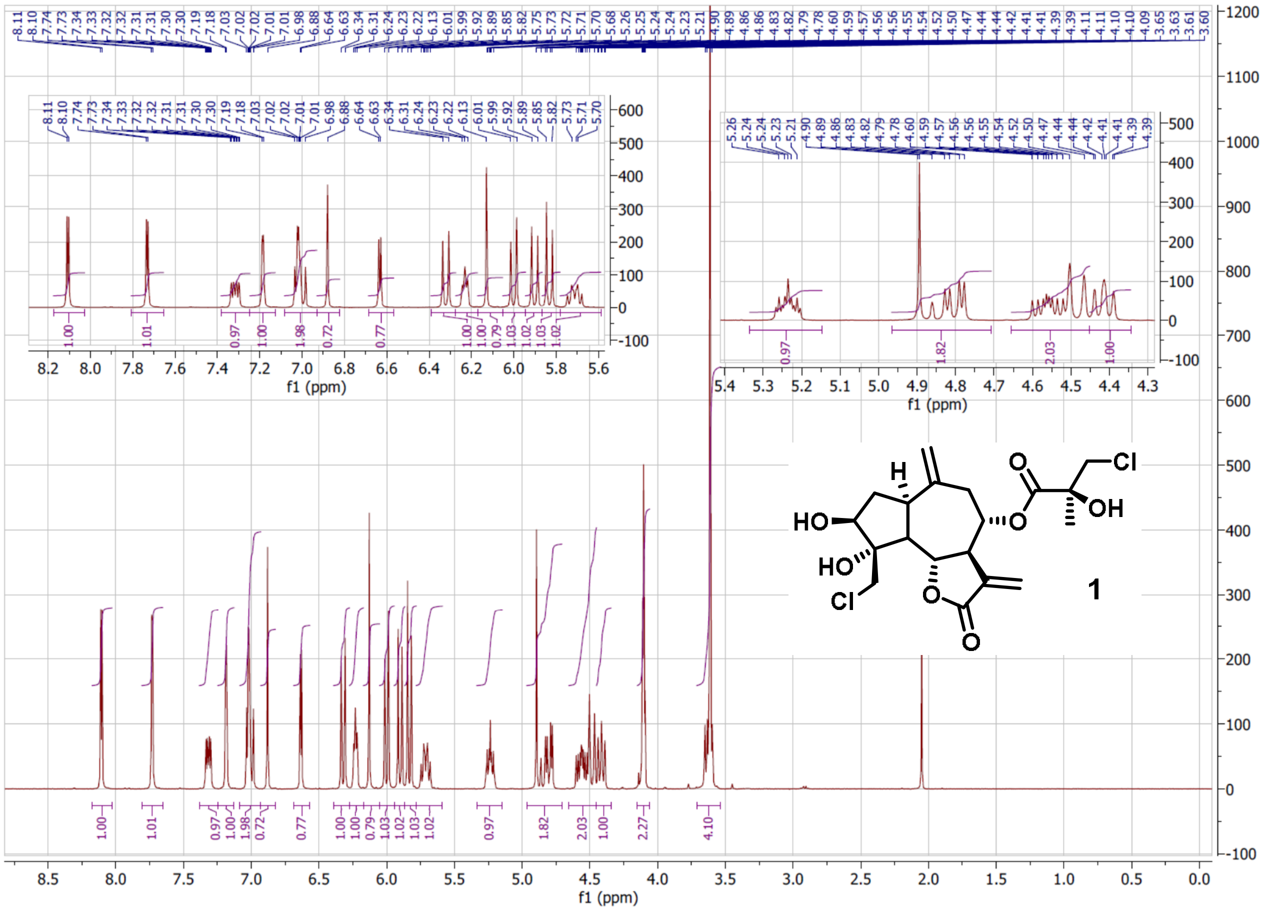

Figure S3: ^{1}H-NMR (500 MHz, Acetone-d6) Spectrum of Compound **1**

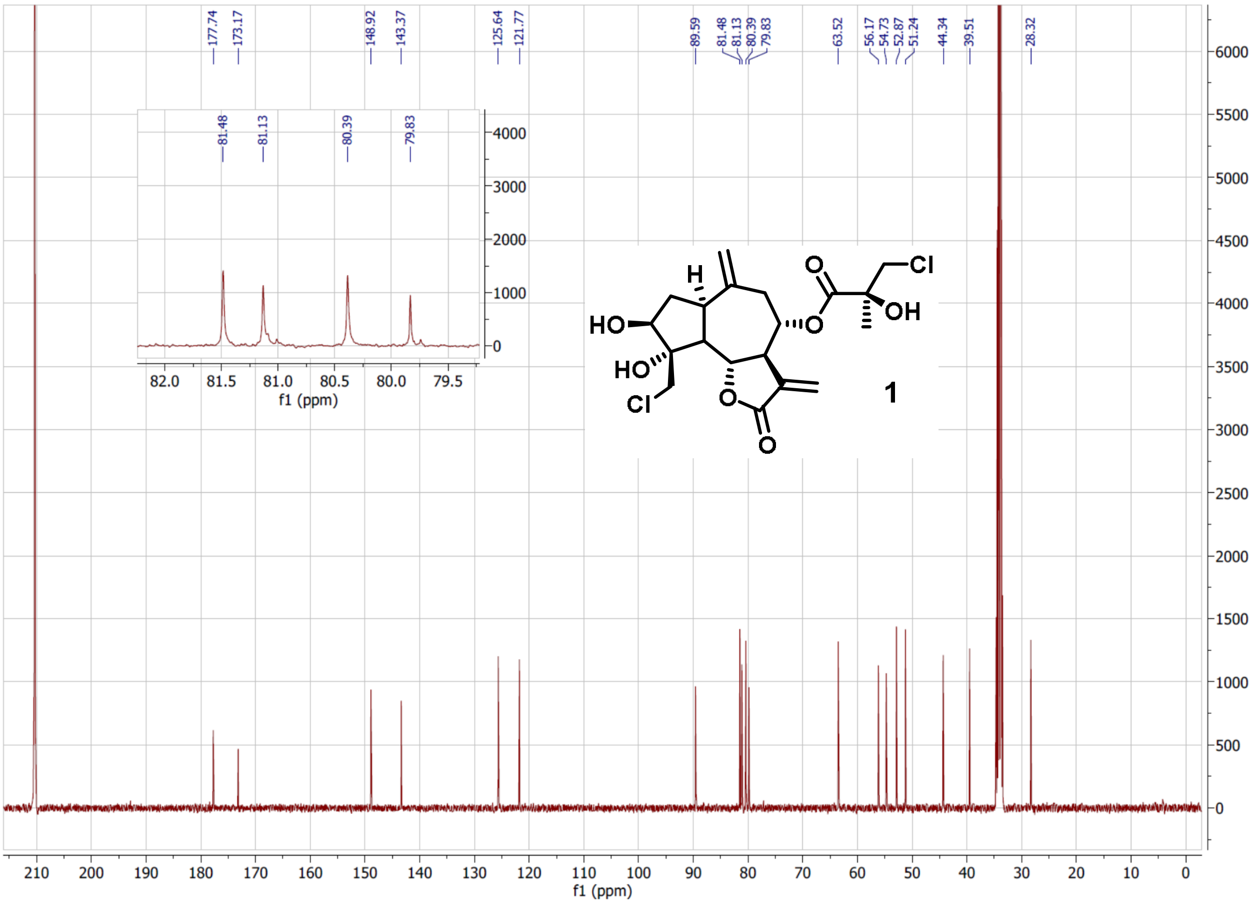

Figure S4: ^{13}C-NMR (125 MHz, Acetone-d6) Spectrum of Compound **1**

Elemental Composition Report **Page 1**

Multiple Mass Analysis: 5 mass(es) processed
Tolerance = 5.0 PPM / DBE: min = -3.0, max = 120.0
Element prediction: Off
Number of isotope peaks used for i-FIT = 3

Monoisotopic Mass, Even Electron Ions
770 formula(e) evaluated with 5 results within limits (all results (up to 1000) for each mass)
Elements Used:
C: 0-19 H: 0-30 O: 0-7 Na: 0-1 35Cl: 0-2 37Cl: 0-2

Ignacio B
(ESI-20-667) Ignacio B (IB-Cl-H-A) 12 (0.487)
1: TOF MS ES+
3.12e+003

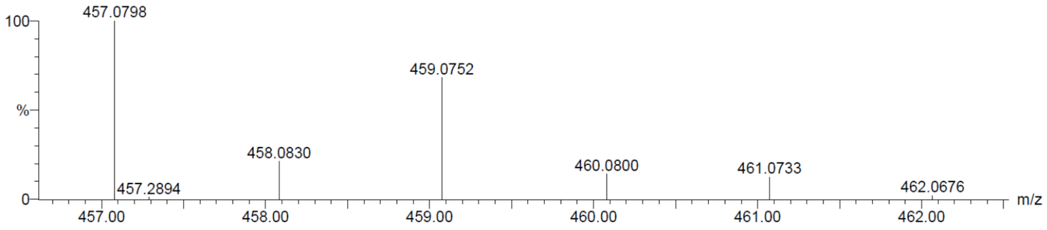

Minimum:	10.00				-3.0			
Maximum:	100.00		5.0	5.0	120.0			
Mass	RA	Calc. Mass	mDa	PPM	DBE	i-FIT	i-FIT (Norm)	Formula
457.0798	100.00	457.0797	0.1	0.2	6.5	35.4	0.0	C19 H24 O7 Na 35Cl2
458.0830	21.17	---						
459.0752	68.12	459.0767	-1.5	-3.3	6.5	20.8	0.9	C19 H24 O7 Na 35Cl 37Cl
		459.0736	1.6	3.5	5.5	20.4	0.5	C19 H26 O6 35Cl 37Cl2
460.0800	14.25	---						
461.0733	12.42	461.0738	-0.5	-1.1	6.5	18.7	1.0	C19 H24 O7 Na 37Cl2
		461.0715	1.8	3.9	4.5	18.2	0.5	C18 H26 O7 35Cl2 37Cl

Figure S5: HRESI-MS Spectrum of Compound **1**

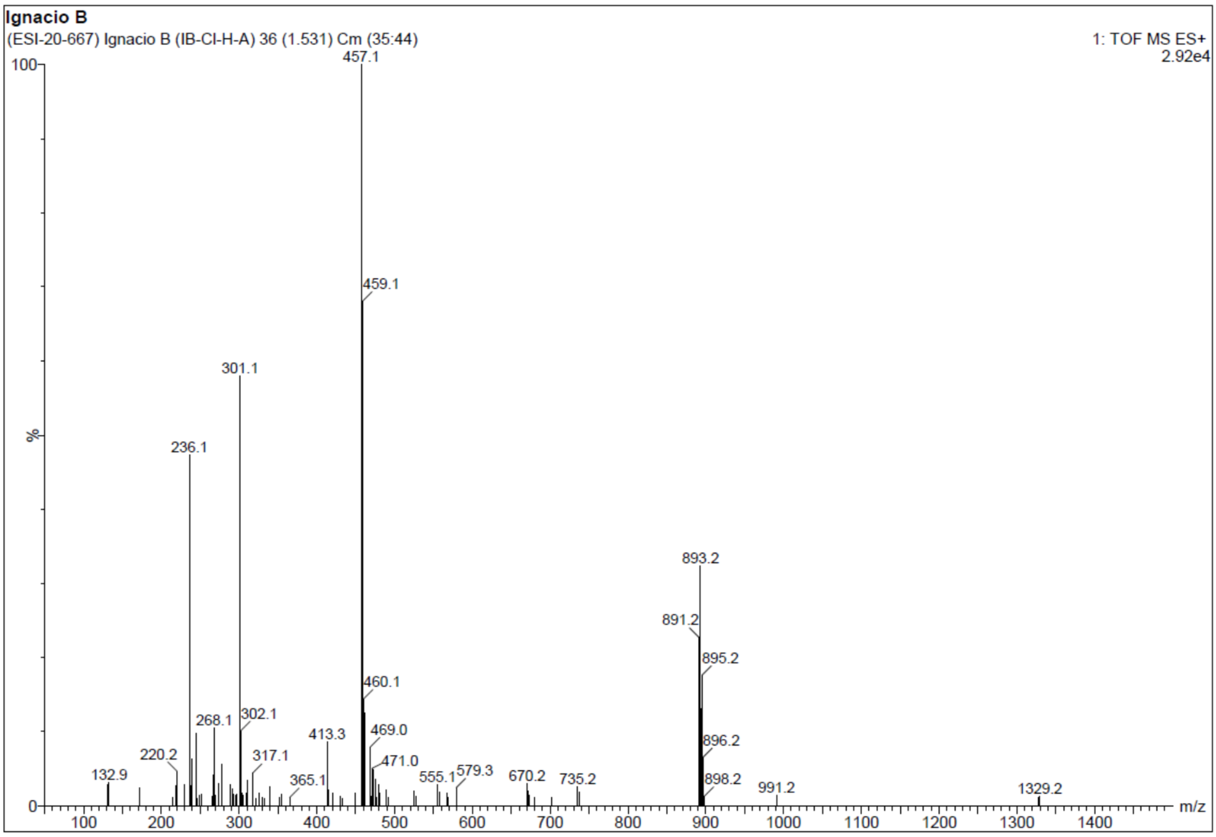

Figure S6: ESI-MS Spectrum of Compound **1**

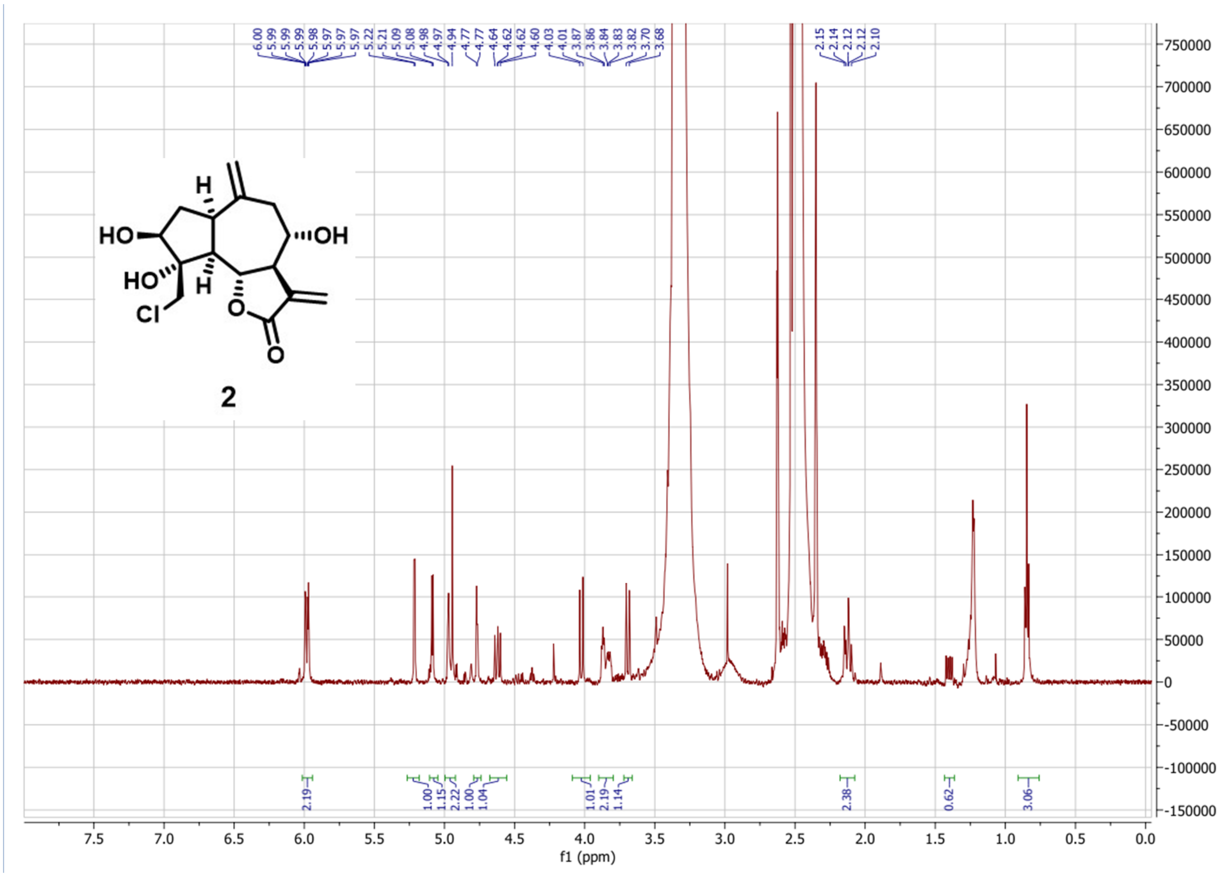

Figure S7: ^{1}H-NMR (500 MHz, DMSO-d6) Spectrum of Compound **2**

Elemental Composition Report **Page 1**

Multiple Mass Analysis: 2 mass(es) processed
Tolerance = 5.0 PPM / DBE: min = -3.0, max = 120.0
Element prediction: Off
Number of isotope peaks used for i-FIT = 3

Monoisotopic Mass, Even Electron Ions
1648 formula(e) evaluated with 7 results within limits (all results (up to 1000) for each mass)
Elements Used:
C: 0-60 H: 0-60 N: 0-1 O: 0-10 Na: 0-2 35Cl: 0-1 37Cl: 0-1

Ignacio B
(ESI-20-945) Igancio B (IB-CH- B) 11 (0.453)
1: TOF MS ES-
4.45e+003

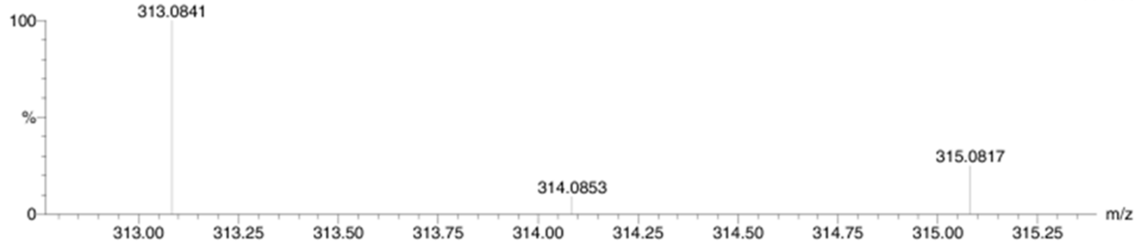

Mass	RA	Calc. Mass	mDa	PPM	DBE	i-FIT	i-FIT (Norm)	Formula
Minimum:	10.00				-3.0			
Maximum:	100.00		5.0	5.0	120.0			
313.0841	100.00	313.0841	0.0	0.0	12.5	26.2	1.9	C19 H14 O3 Na
		313.0843	-0.2	-0.6	6.5	25.6	1.3	C15 H18 O5 35Cl
		313.0844	-0.3	-1.0	-0.5	24.9	0.6	C10 H21 O7 Na 37Cl
315.0817	24.80	315.0821	-0.4	-1.3	4.5	23.9	1.3	C13 H17 O6 Na2
		315.0813	0.4	1.3	6.5	24.1	1.4	C15 H18 O5 37Cl
		315.0823	-0.6	-1.9	-1.5	23.7	1.1	C9 H21 O8 Na 35Cl
		315.0810	0.7	2.2	19.5	24.7	2.0	C24 H11 O

Figure S8: HRESI-MS Spectrum of Compound **2**

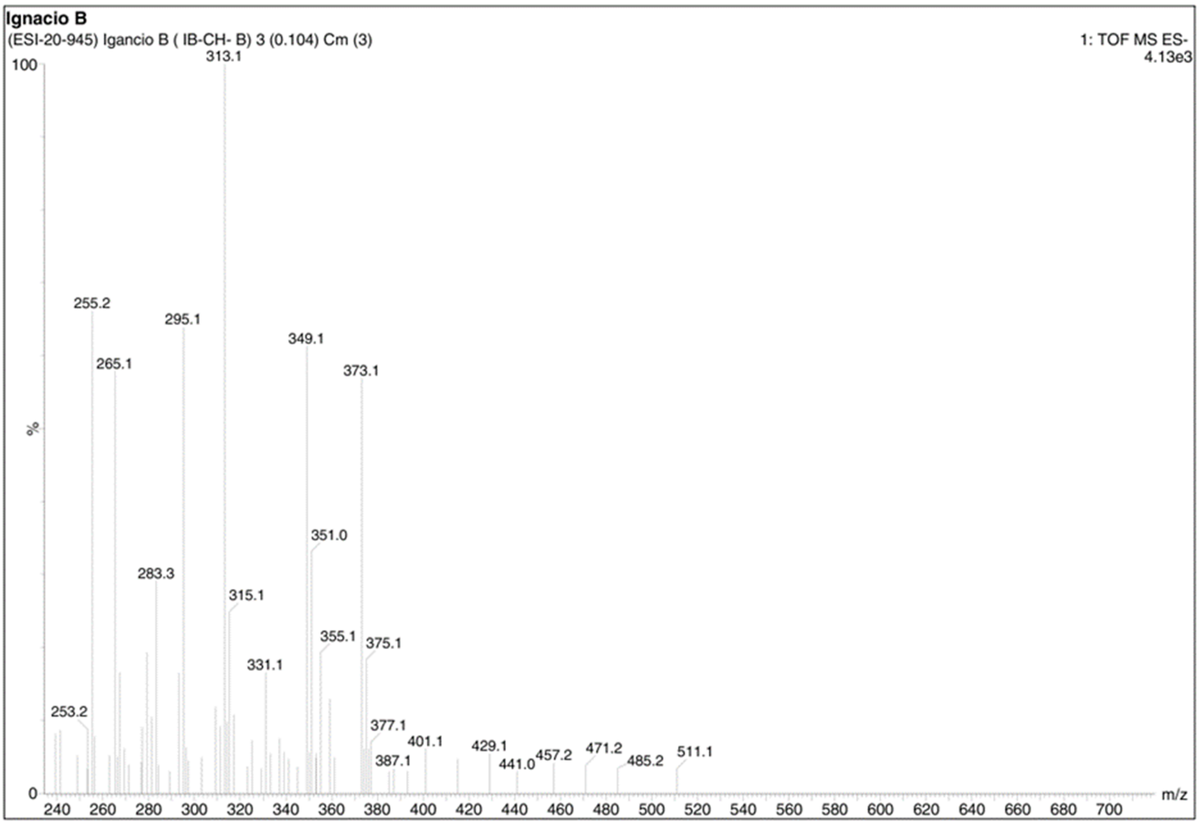

Figure S9: ESI-MS Spectrum of Compound **2**

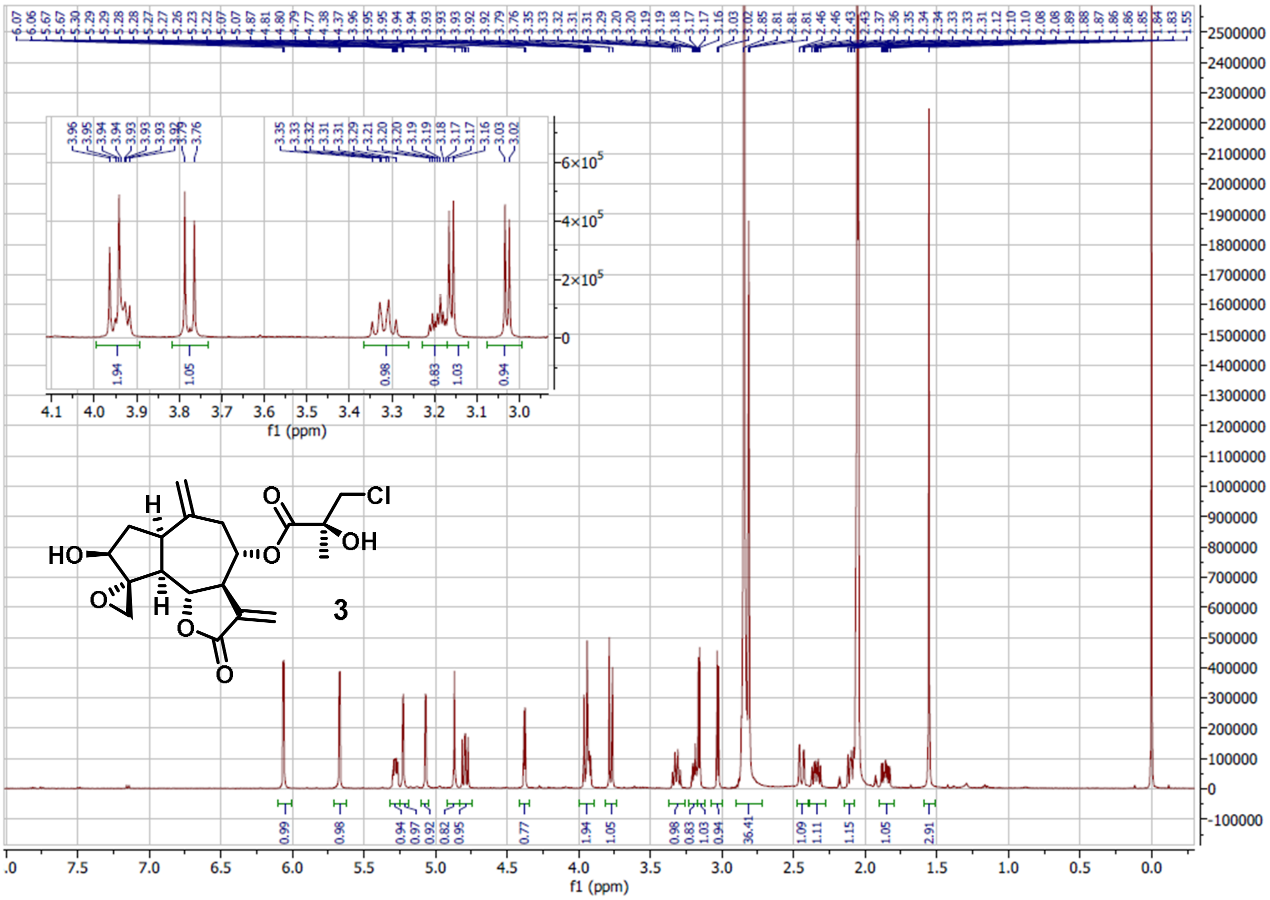

Figure S10: ^{1}H-NMR (500 MHz, Acetone-d6) Spectrum of Compound **3**

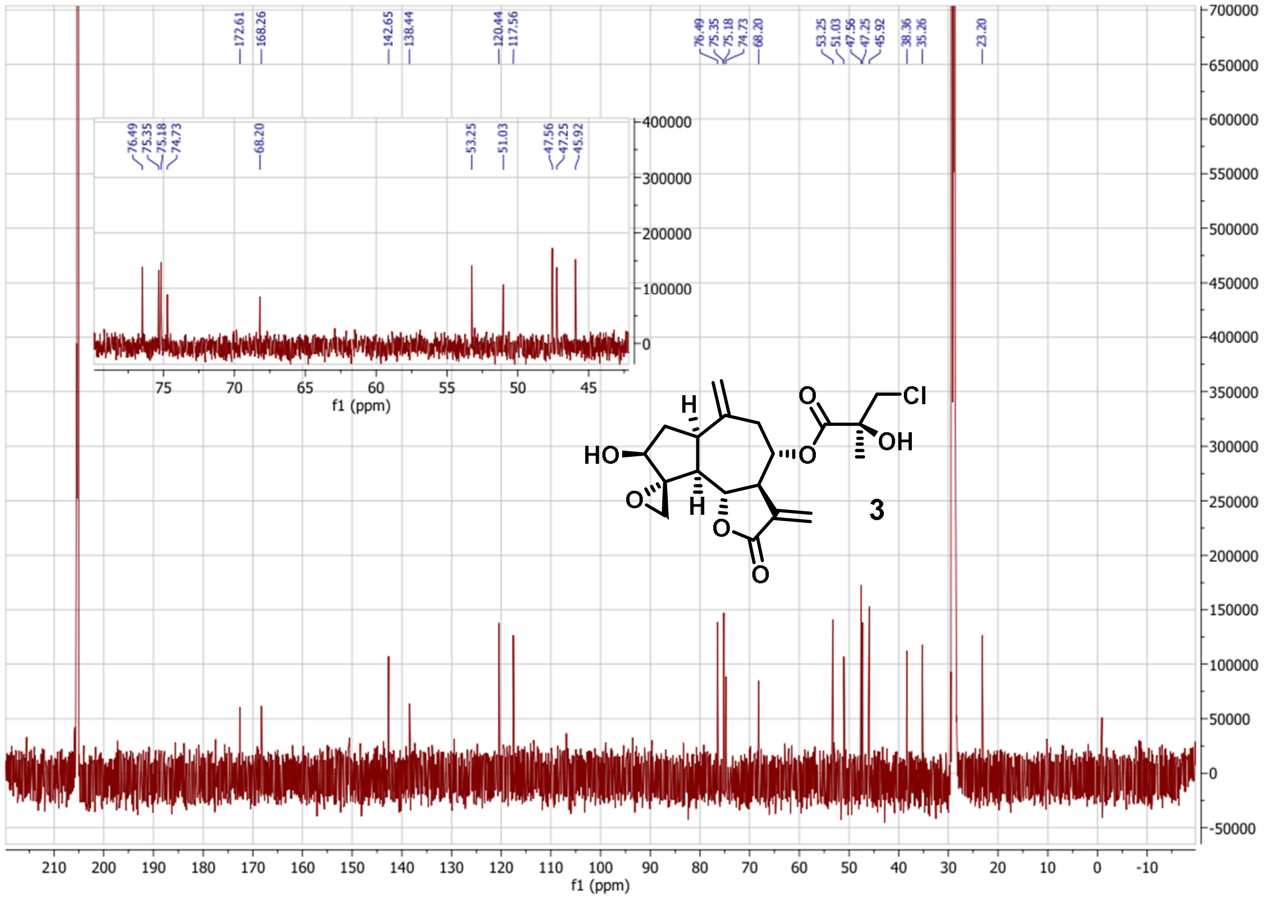

Figure S11: ^{13}C-NMR (125 MHz, Acetone-d6) Spectrum of Compound **3**

Elemental Composition Report

Page 1

Multiple Mass Analysis: 3 mass(es) processed

Tolerance = 5.0 PPM / DBE: min = -3.0, max = 120.0
Element prediction: Off
Number of isotope peaks used for i-FIT = 3

Monoisotopic Mass, Even Electron Ions
443 formula(e) evaluated with 2 results within limits (all results (up to 1000) for each mass)
Elements Used:
C: 0-23 H: 0-100 O: 0-7 Na: 0-1 35Cl: 0-1 37Cl: 0-1

Ignacio B
(ESI-20-693) Ignacio B (IB CL-H-C) 21 (0.678)

2: TOF MS ES+
9.16e+002

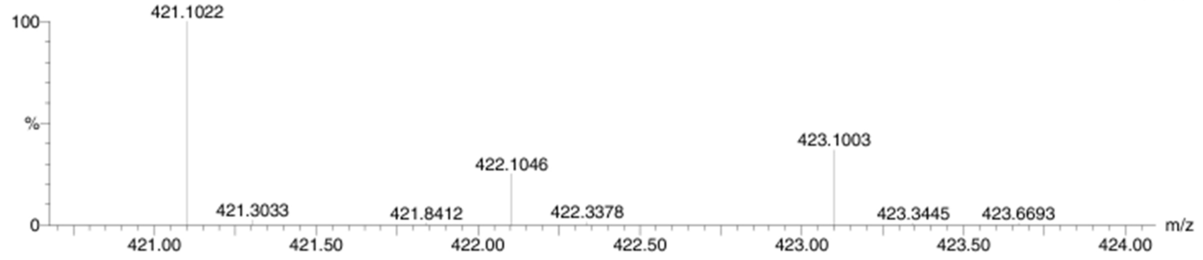

Mass	RA	Calc. Mass	mDa	PPM	DBE	i-FIT	i-FIT (Norm)	Formula
Minimum:	23.00				-3.0			
Maximum:	100.00		5.0	5.0	120.0			
421.1022	100.00	421.1030	-0.8	-1.9	7.5	41.3	0.0	C19 H23 O7 Na 35Cl
422.1046	25.15	---						
423.1003	36.78	423.1001	0.2	0.5	7.5	31.3	0.0	C19 H23 O7 Na 37Cl

Figure S12: HRESI-MS Spectrum of Compound **3**

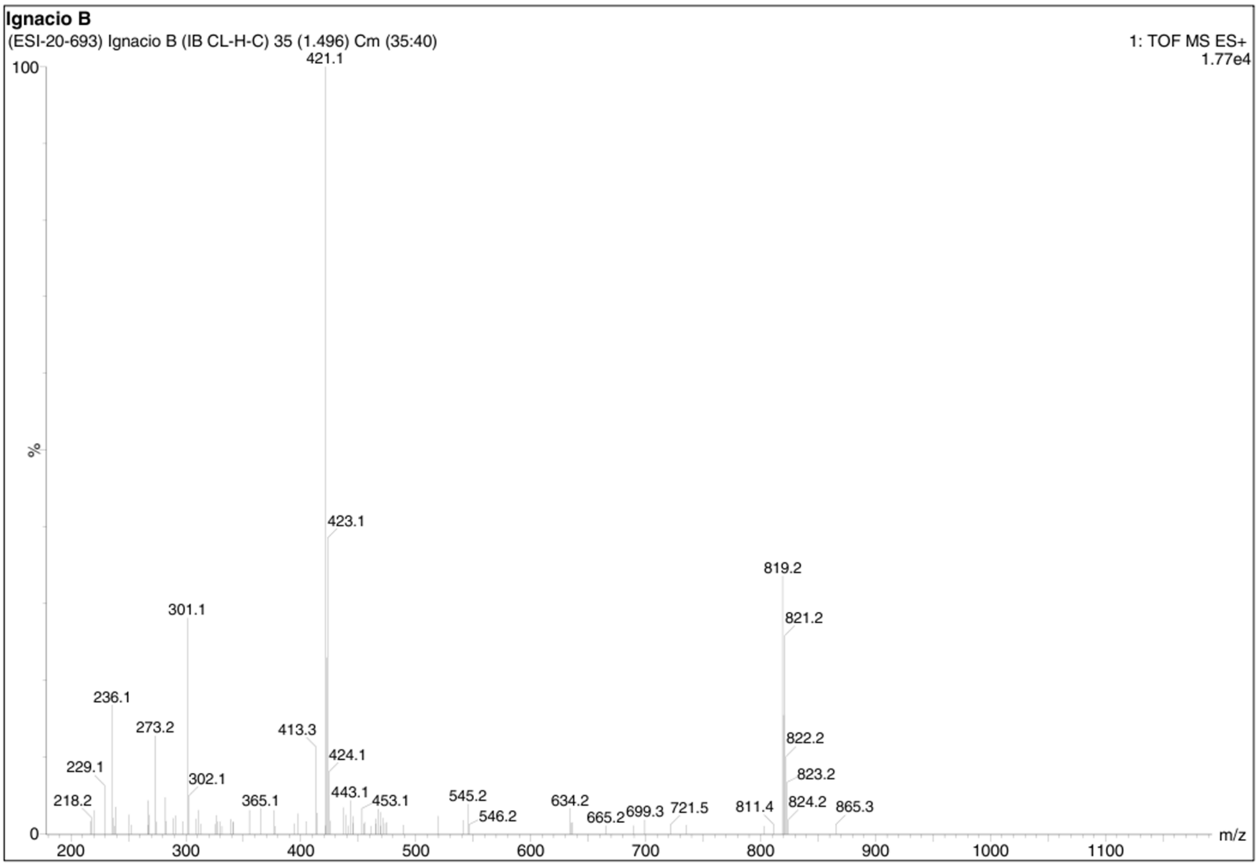

Figure S13: ESI-MS Spectrum of Compound **3**

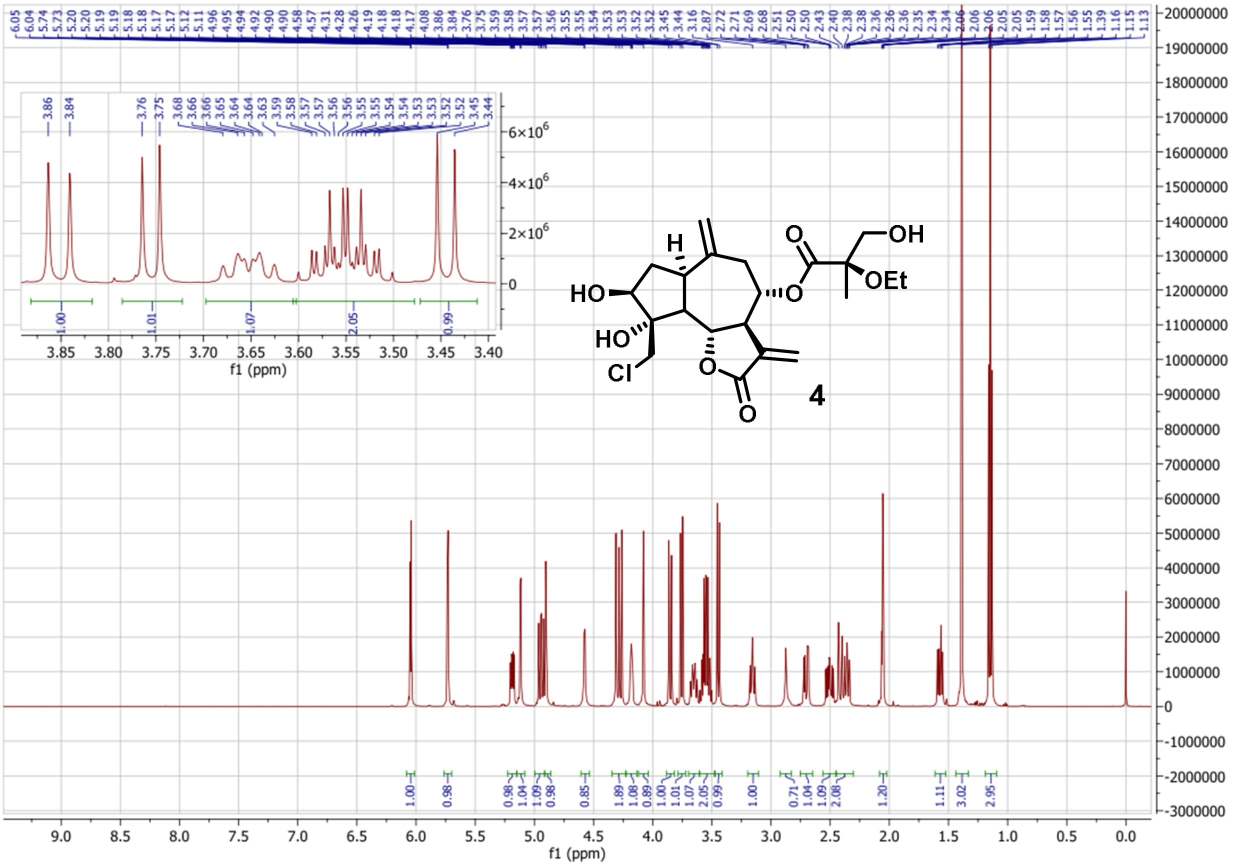

Figure S14: ^{1}H-NMR (500 MHz, Acetone-d6) Spectrum of Compound **4**

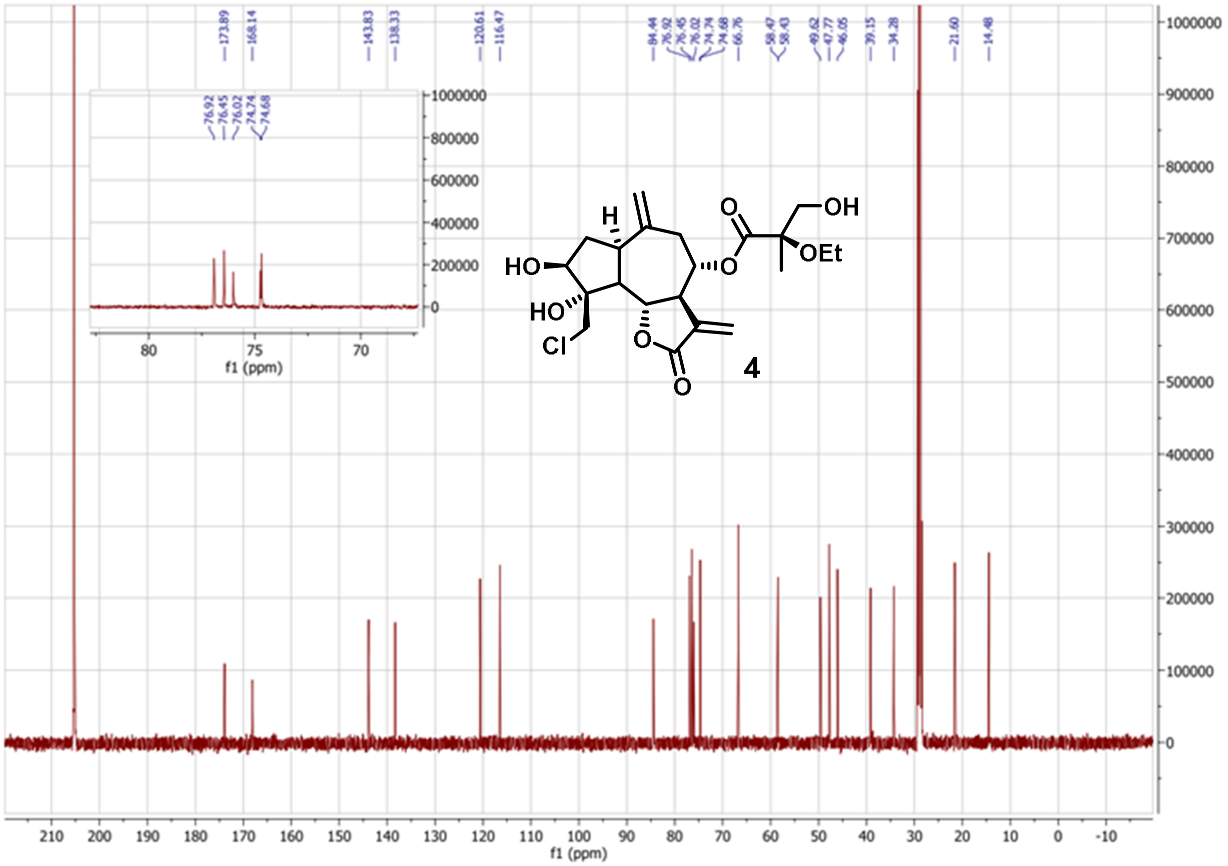

Figure S15: ^{13}C-NMR (125 MHz, Acetone-d6) Spectrum of Compound **4**

Elemental Composition Report **Page 1**

Multiple Mass Analysis: 3 mass(es) processed
Tolerance = 5.0 PPM / DBE: min = -3.0, max = 120.0
Element prediction: Off
Number of isotope peaks used for i-FIT = 3

Monoisotopic Mass, Even Electron Ions
460 formula(e) evaluated with 3 results within limits (all results (up to 1000) for each mass)
Elements Used:
C: 0-25 H: 0-60 O: 0-8 Na: 0-1 35Cl: 0-1 37Cl: 0-1
Ignacio B
(ESI-20-708) Ignacio B (IB Cl H D) 55 (1.861) 2: TOF MS ES+ 5.36e+003

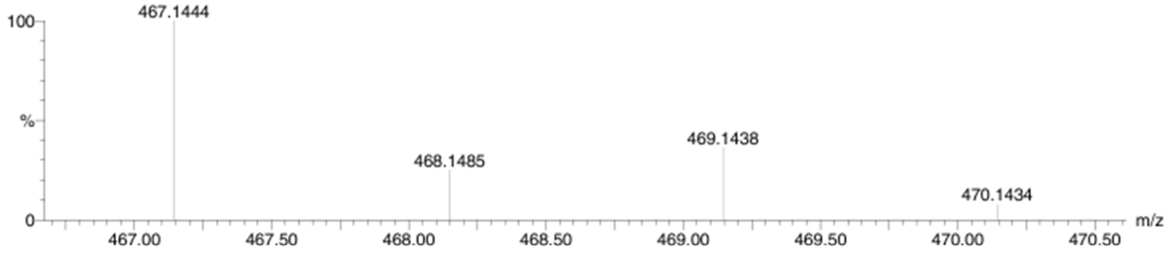

Mass	RA	Calc. Mass	mDa	PPM	DBE	i-FIT	i-FIT (Norm)	Formula
Minimum:	23.00				-3.0			
Maximum:	100.00		5.0	5.0	120.0			
467.1444	100.00	467.1449	-0.5	-1.1	6.5	25.9	0.0	C21 H29 O8 Na 35Cl
468.1485	24.89	---						
469.1438	36.19	469.1443	-0.5	-1.1	9.5	24.5	0.9	C23 H28 O8 37Cl
		469.1419	1.9	4.0	6.5	24.2	0.6	C21 H29 O8 Na 37Cl

Figure S16: HRESI-MS Spectrum of Compound **4**

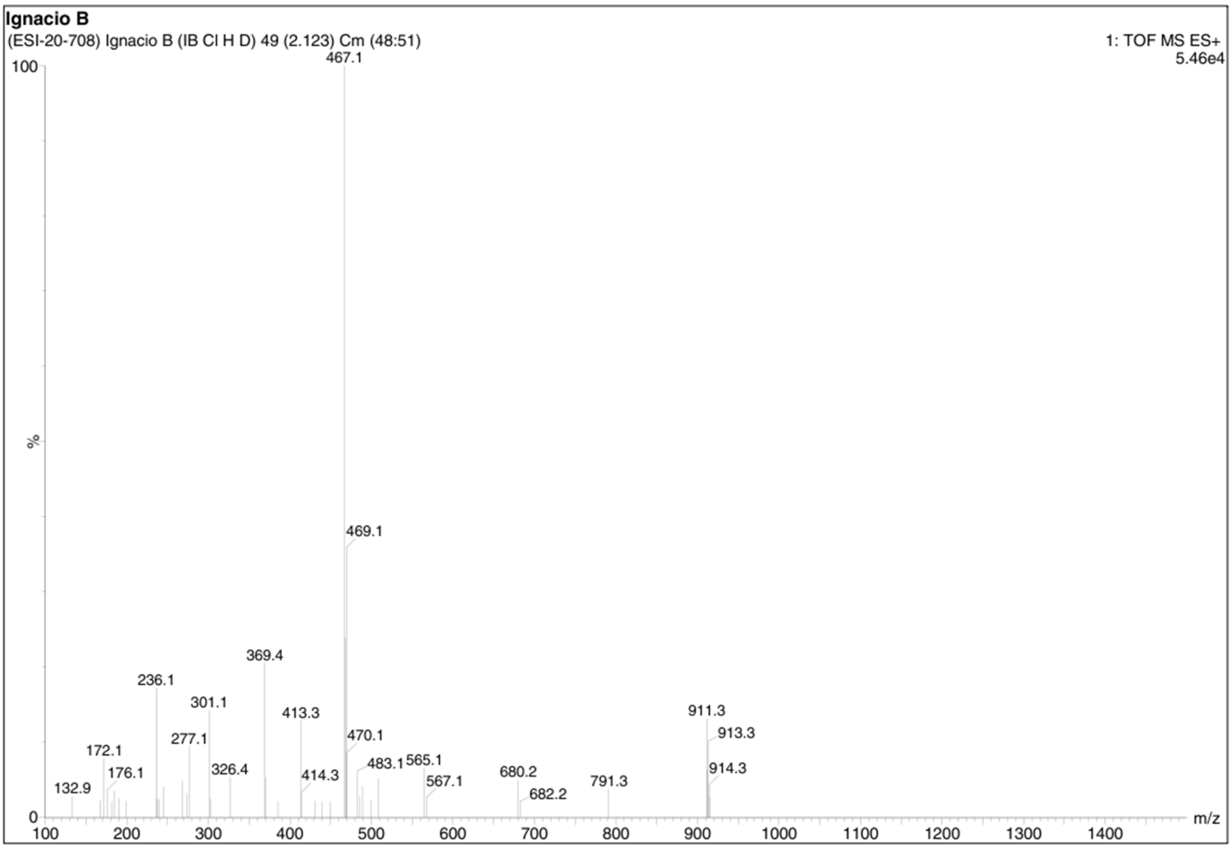

Figure S17: ESI-MS Spectrum of Compound **4**

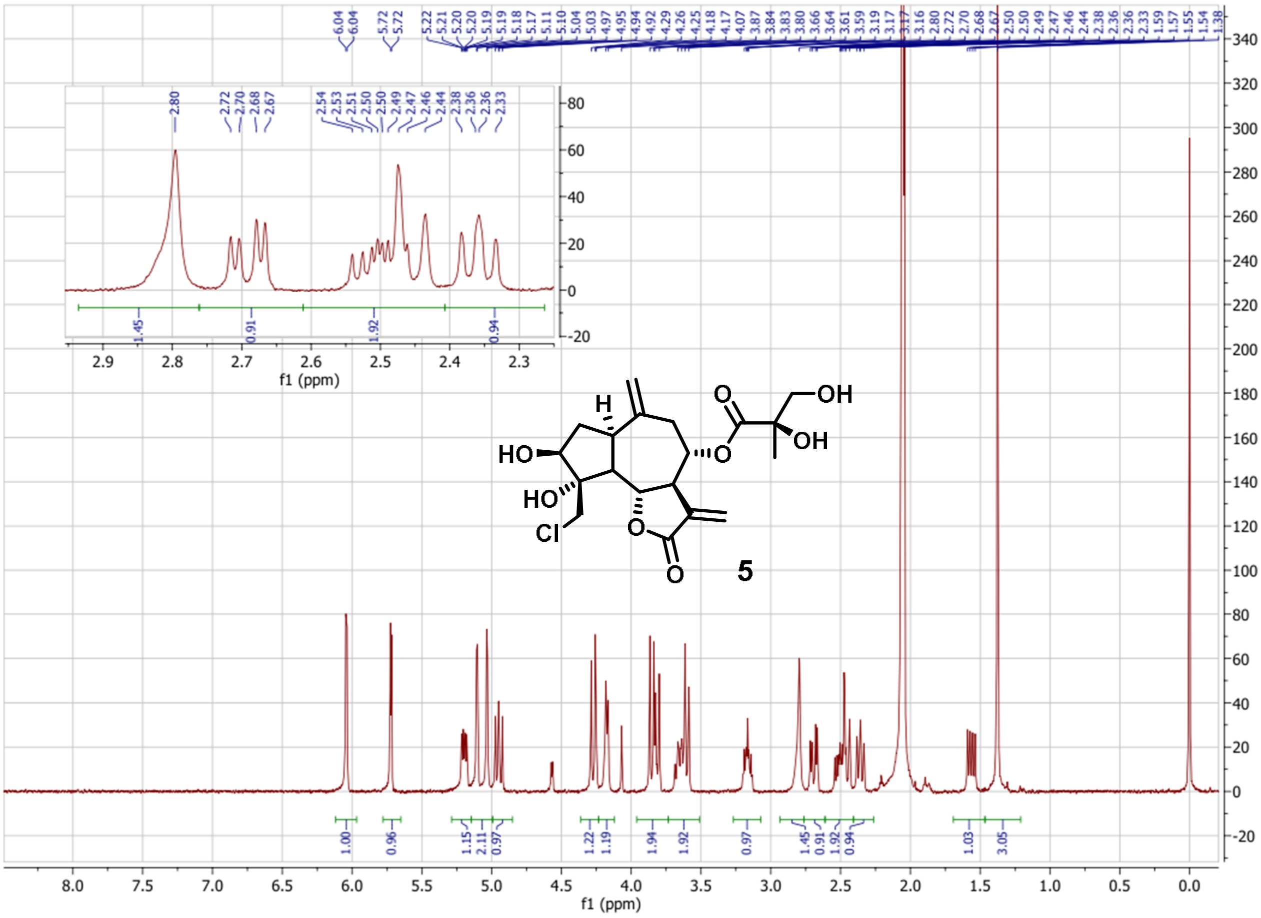

Figure S18: ^{1}H-NMR (500 MHz, Acetone-d6) Spectrum of Compound **5**

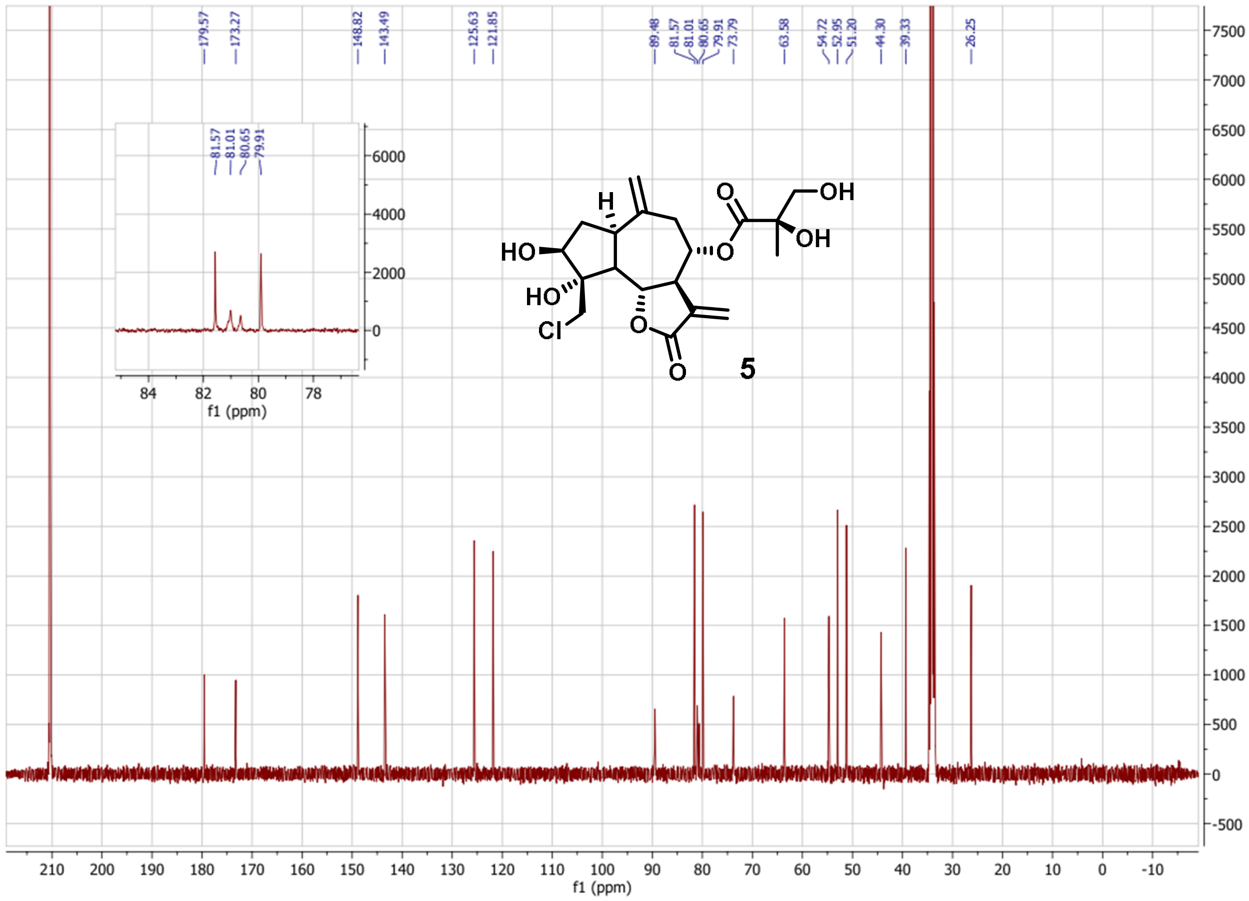

Figure S19: ^{13}C-NMR (125 MHz, Acetone-d6) Spectrum of Compound **5**

Elemental Composition Report **Page 1**

Multiple Mass Analysis: 4 mass(es) processed
Tolerance = 5.0 PPM / DBE: min = -3.0, max = 120.0
Element prediction: Off
Number of isotope peaks used for i-FIT = 3

Monoisotopic Mass, Even Electron Ions
803 formula(e) evaluated with 3 results within limits (all results (up to 1000) for each mass)
Elements Used:
C: 0-20 H: 0-52 O: 0-12 Na: 0-1 35Cl: 0-1 37Cl: 0-1

Ignacio B
(ESI-20-678) Ignacio B (IB CL H E) 20 (0.834) 1: TOF MS ES+ 4.67e+003

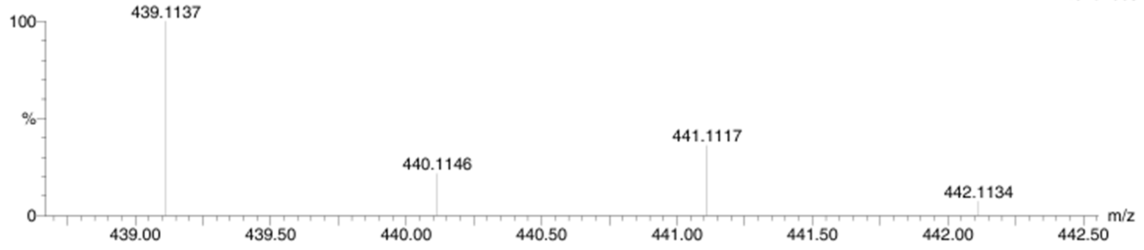

Minimum:	5.00				-3.0			
Maximum:	100.00		5.0	5.0	120.0			
Mass	RA	Calc. Mass	mDa	PPM	DBE	i-FIT	i-FIT (Norm)	Formula
439.1137	100.00	439.1136	0.1	0.2	6.5	25.9	0.0	C19 H25 O8 Na 35Cl
440.1146	21.61	---						
441.1117	36.01	441.1108	0.9	2.0	0.5	21.5	0.6	C15 H29 O10 35Cl 37Cl
		441.1106	1.1	2.5	6.5	21.8	0.8	C19 H25 O8 Na 37Cl
442.1134	7.28	---						

Figure S20: HRESI-MS Spectrum of Compound **5**

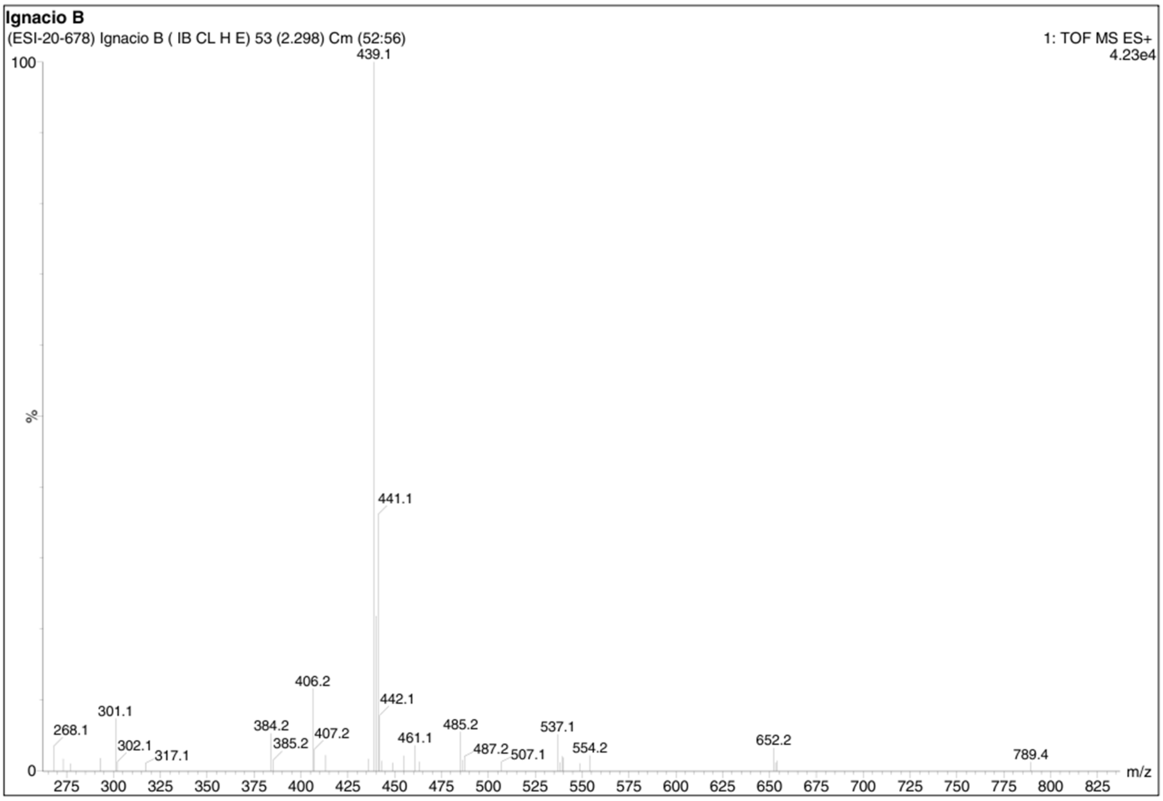

Figure S21: ESI-MS Spectrum of Compound **5**

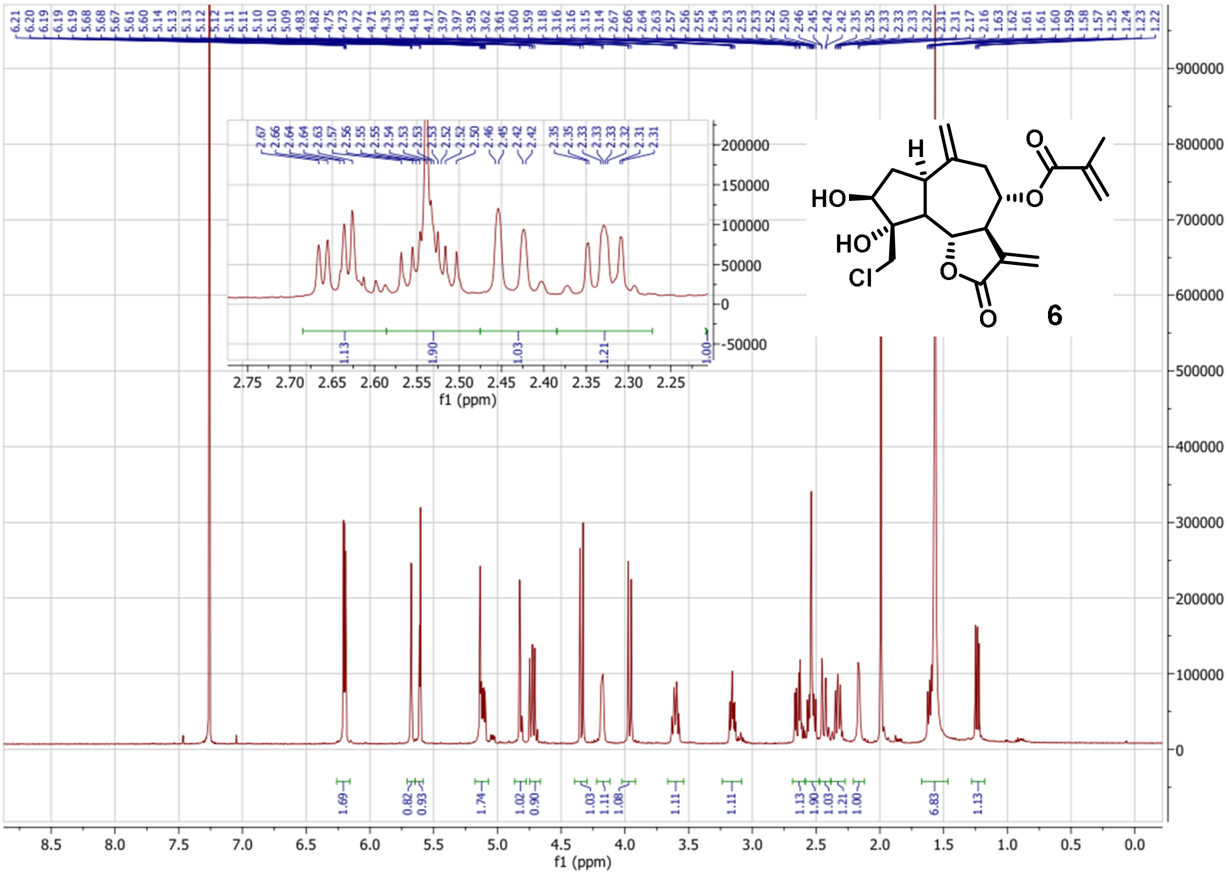

Figure S22: ^{1}H-NMR (500 MHz, $CDCl_3$) Spectrum of Compound **6**

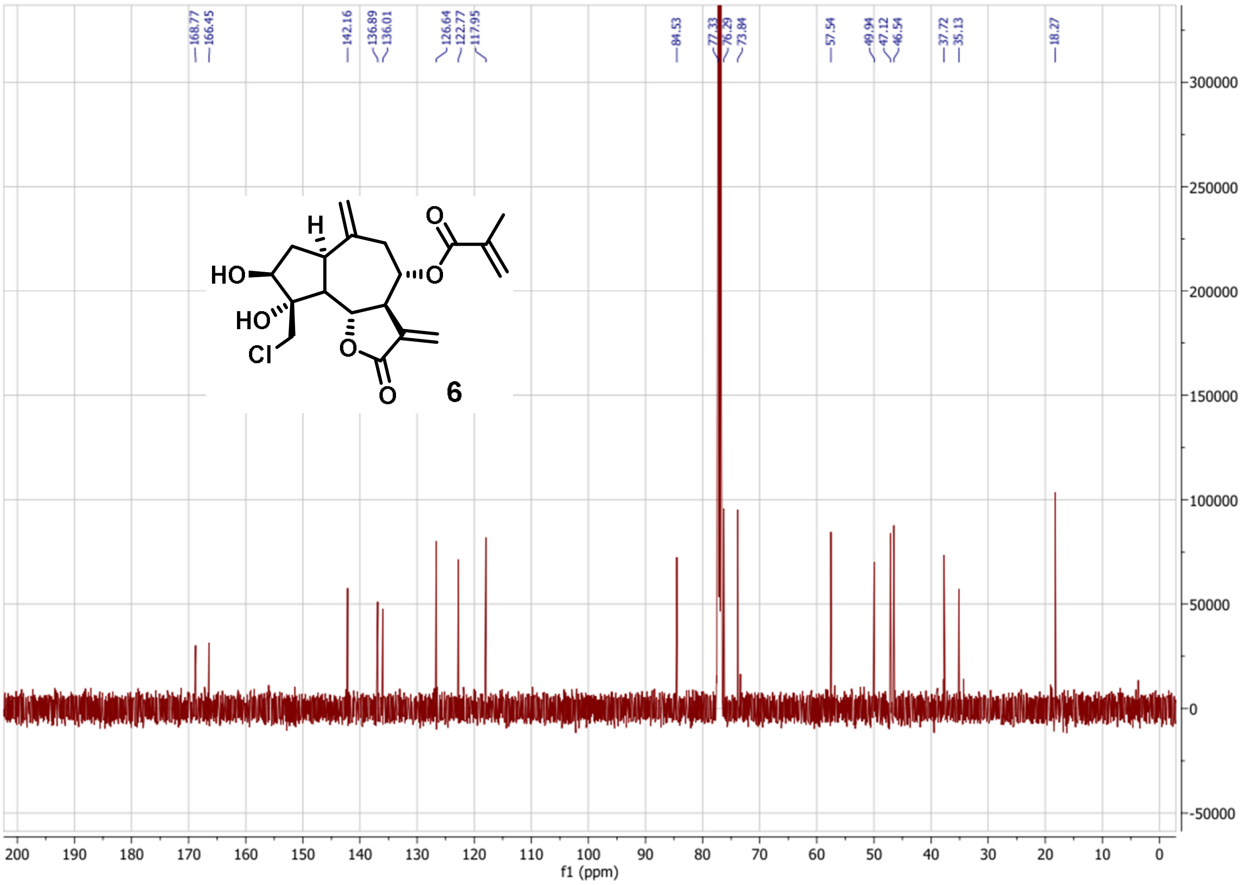

Figure S23: ^{13}C-NMR (125 MHz, $CDCl_3$) Spectrum of Compound **6**

Elemental Composition Report

Page 1

Multiple Mass Analysis: 3 mass(es) processed

Tolerance = 5.0 PPM / DBE: min = -3.0, max = 120.0
Element prediction: Off
Number of isotope peaks used for i-FIT = 3

Monoisotopic Mass, Even Electron Ions
247 formula(e) evaluated with 3 results within limits (all results (up to 1000) for each mass)
Elements Used:
C: 0-20 H: 0-60 O: 0-6 Na: 0-1 35Cl: 0-1 37Cl: 0-1
Ignacio B
(ESI-20-707) Ignacio B (IB-Lin -A) 82 (3.551)

1: TOF MS ES+
8.20e+002

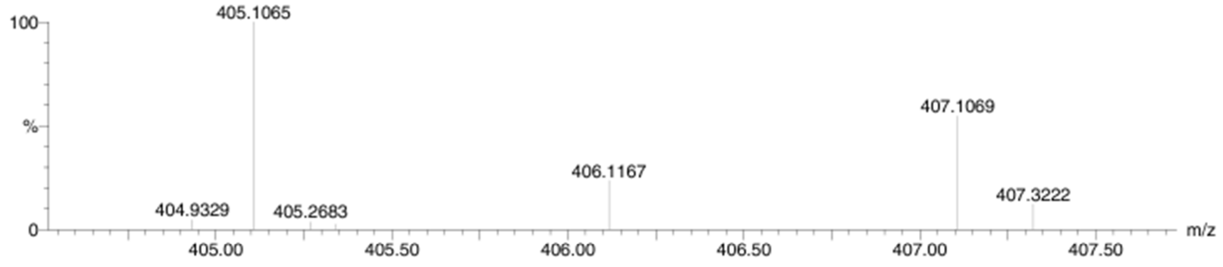

Minimum:	23.00				-3.0			
Maximum:	100.00		5.0	5.0	120.0			
Mass	RA	Calc. Mass	mDa	PPM	DBE	i-FIT	i-FIT (Norm)	Formula
405.1065	100.00	405.1050	1.5	3.7	6.5	43.7	0.6	C19 H25 O5 35Cl 37Cl
		405.1081	-1.6	-3.9	7.5	44.0	0.8	C19 H23 O6 Na 35Cl
406.1167	23.44	---						
407.1069	54.60	407.1051	1.8	4.4	7.5	32.3	0.0	C19 H23 O6 Na 37Cl

Figure S24: HRESI-MS Spectrum of Compound **6**

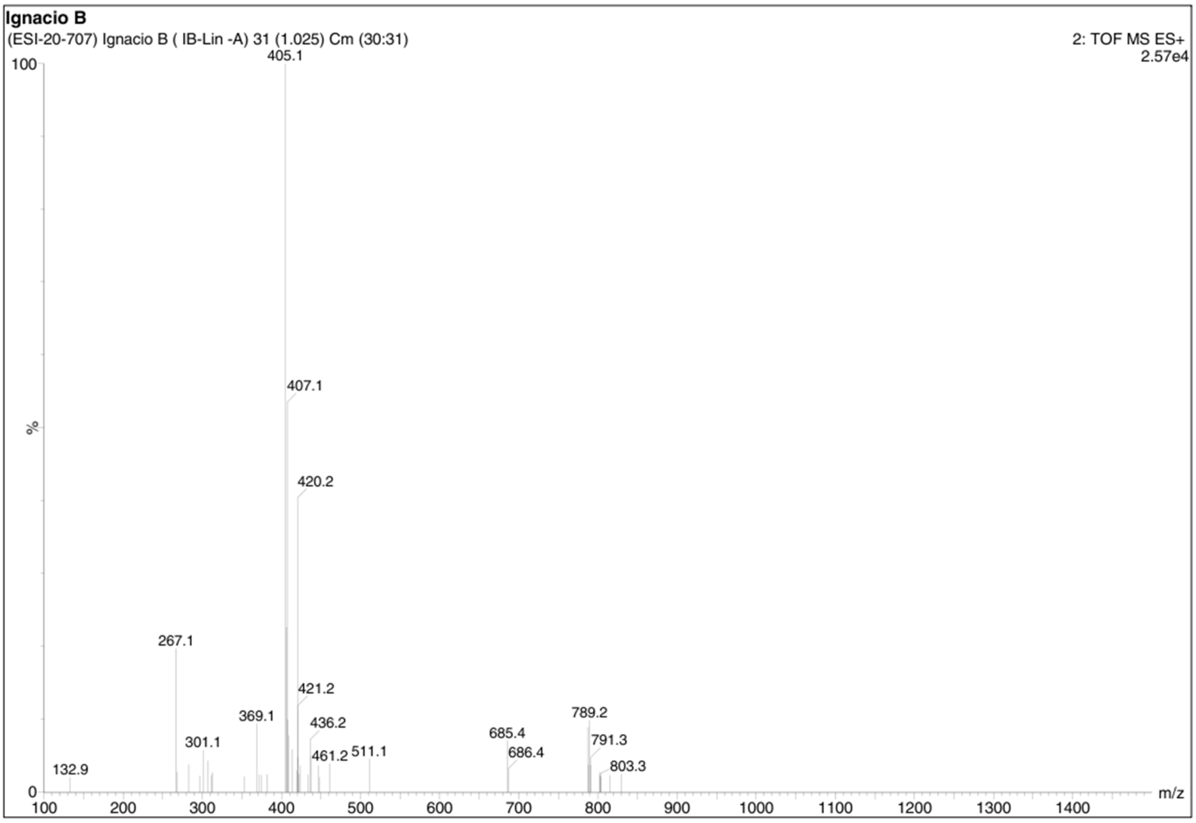

Figure S25: ESI-MS Spectrum of Compound **6**

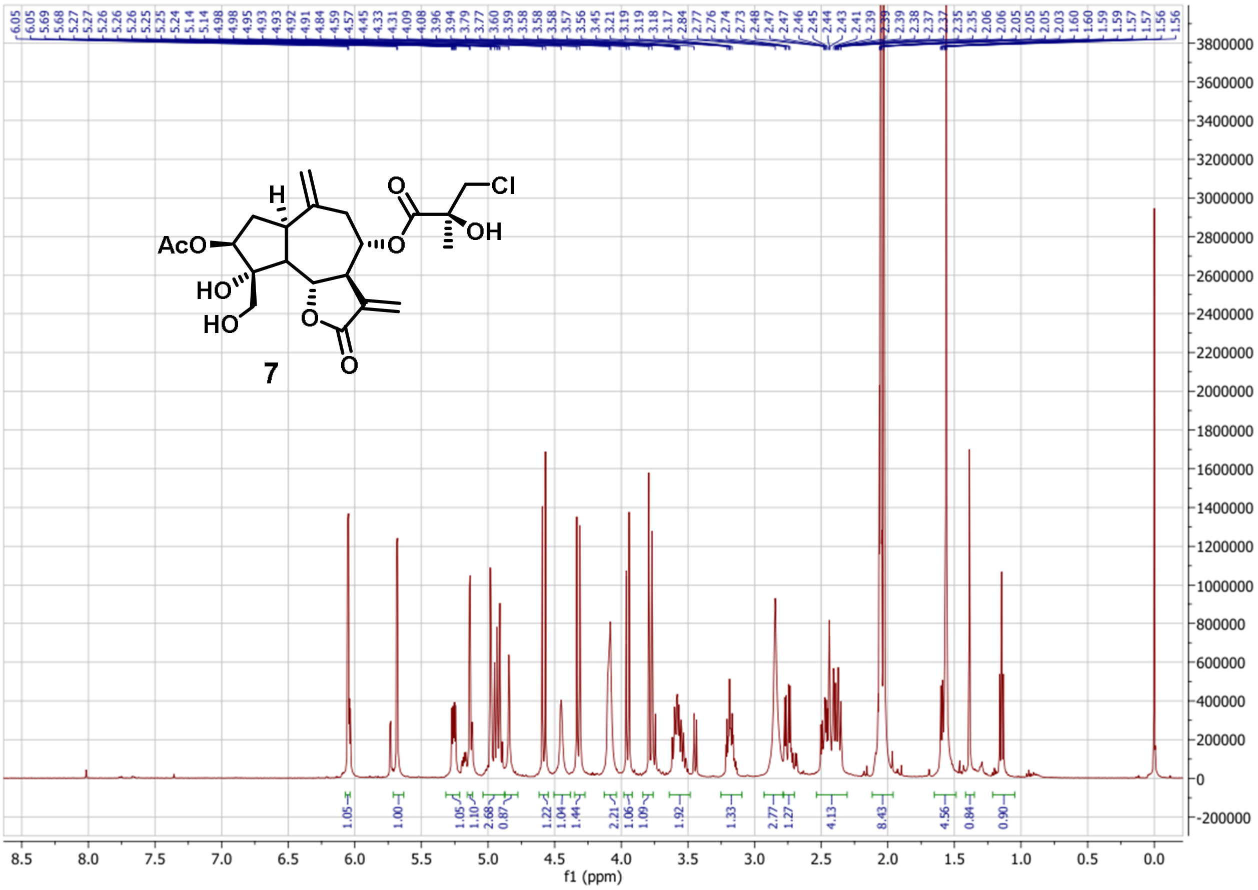

Figure S26: ^{1}H-NMR (500 MHz, Acetone-d6) Spectrum of Compound **7**

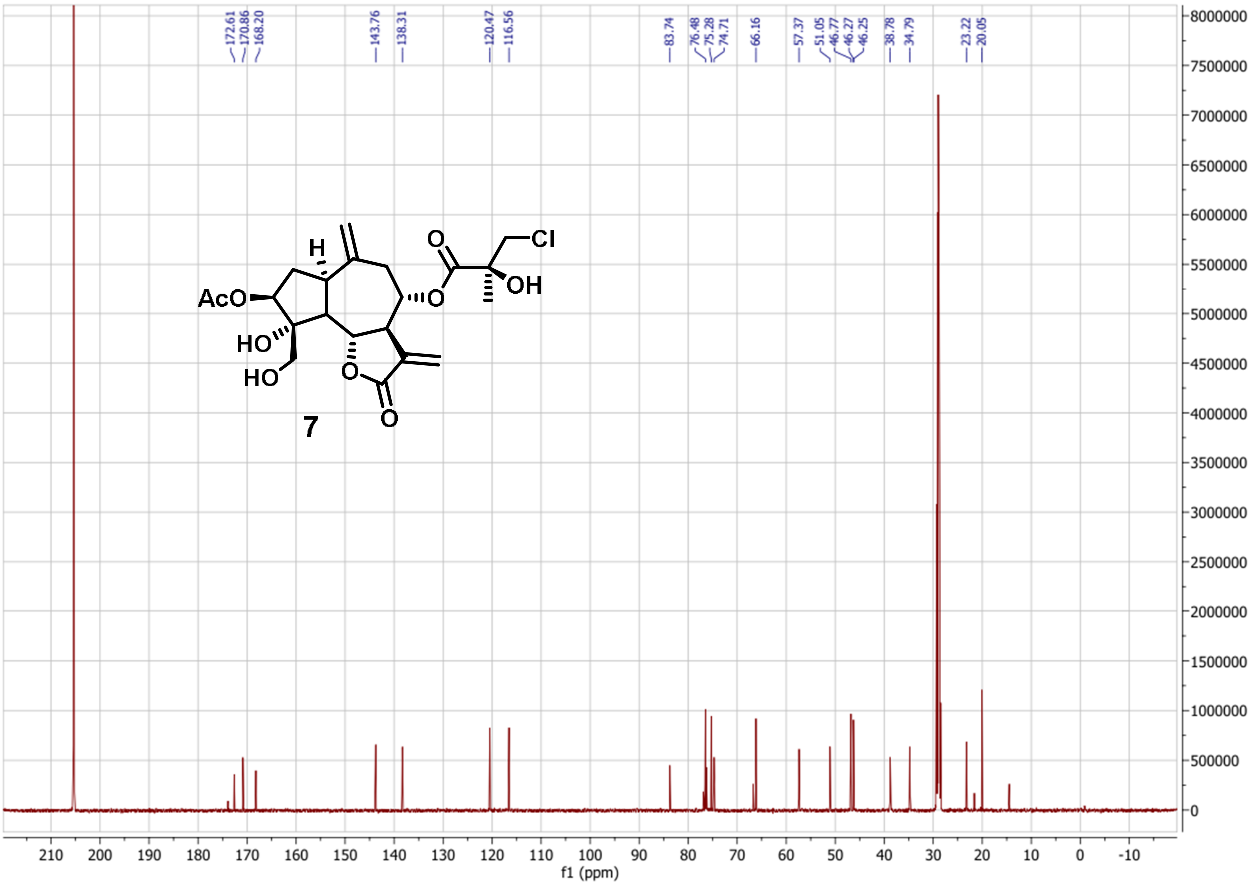

Figure S27: ^{13}C-NMR (125 MHz, Acetone-d6) Spectrum of Compound **7**

Elemental Composition Report **Page 1**

Multiple Mass Analysis: 4 mass(es) processed
Tolerance = 5.0 PPM / DBE: min = -3.0, max = 120.0
Element prediction: Off
Number of isotope peaks used for i-FIT = 3

Monoisotopic Mass, Even Electron Ions
525 formula(e) evaluated with 2 results within limits (all results (up to 1000) for each mass)
Elements Used:
C: 0-22 H: 0-60 O: 0-10 Na: 0-1 35Cl: 0-1 37Cl: 0-1
Ignacio B
(ESI-20-736) Ignacio B (IB -LIN -C) 24 (1.009)

1: TOF MS ES+
6.49e+003

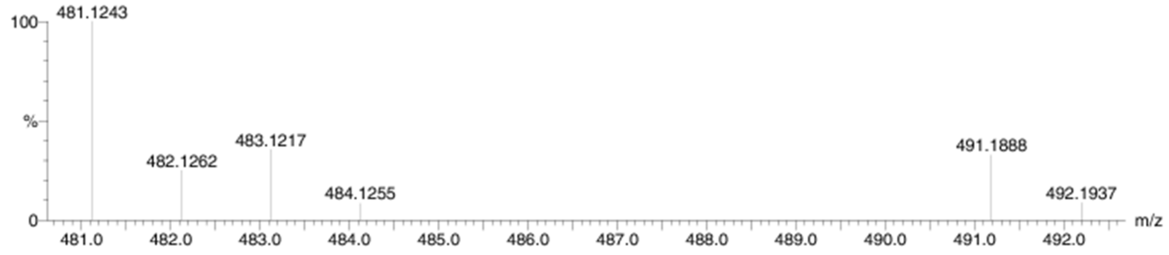

Minimum:	20.00				-3.0			
Maximum:	100.00		5.0	5.0	120.0			
Mass	RA	Calc. Mass	mDa	PPM	DBE	i-FIT	i-FIT (Norm)	Formula
481.1243	100.00	481.1241	0.2	0.4	7.5	26.7	0.0	C21 H27 O9 Na 35Cl
482.1262	24.85	---						
483.1217	35.43	483.1212	0.5	1.0	7.5	23.1	0.0	C21 H27 O9 Na 37Cl
491.1888	32.62	---						

Figure S28: HRESI-MS Spectrum of Compound **7**

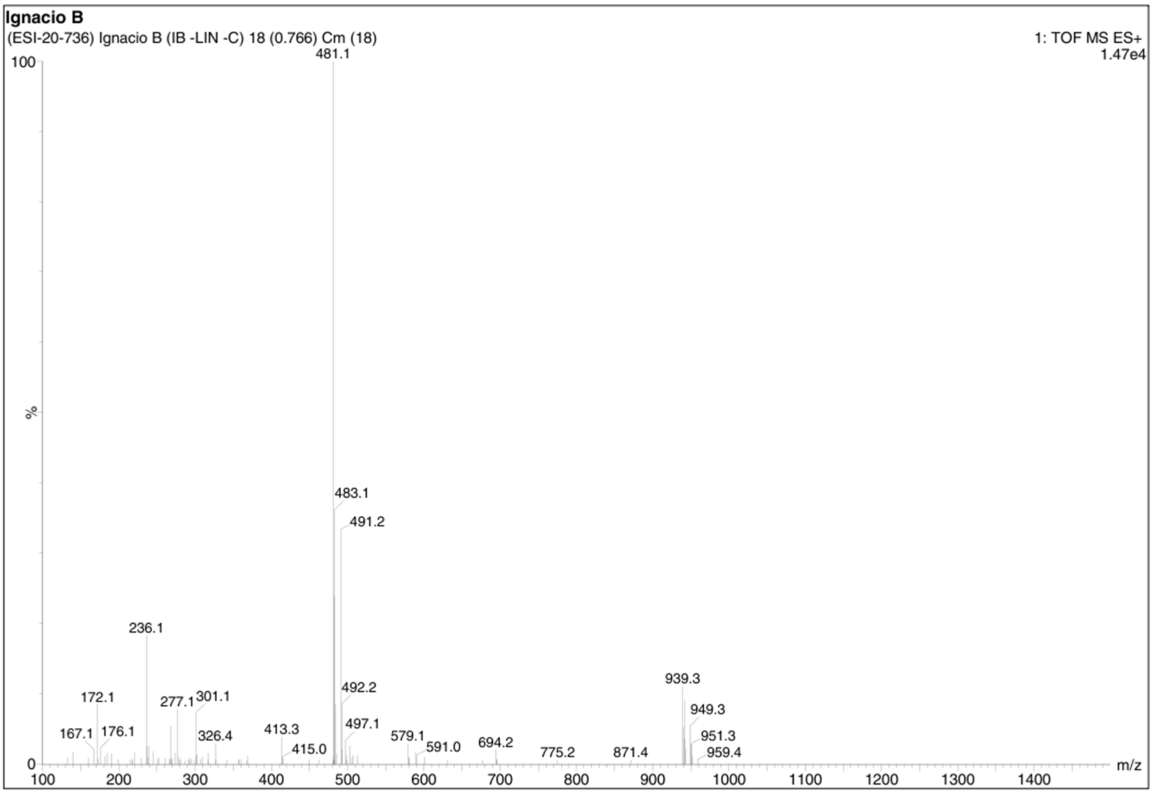

Figure S29: ESI-MS Spectrum of Compound **7**

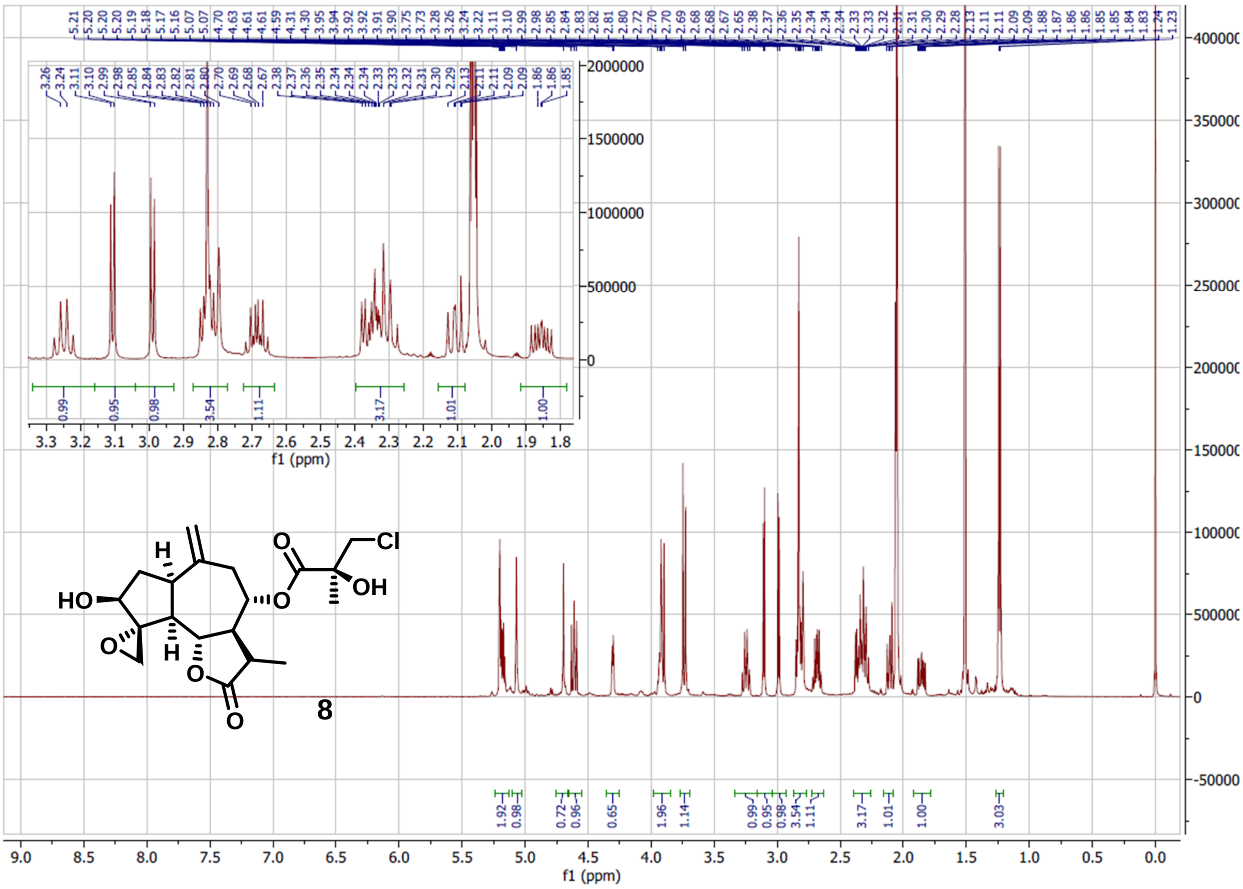

Figure S30: ^{1}H-NMR (500 MHz, Acetone-d6) Spectrum of Compound **8**

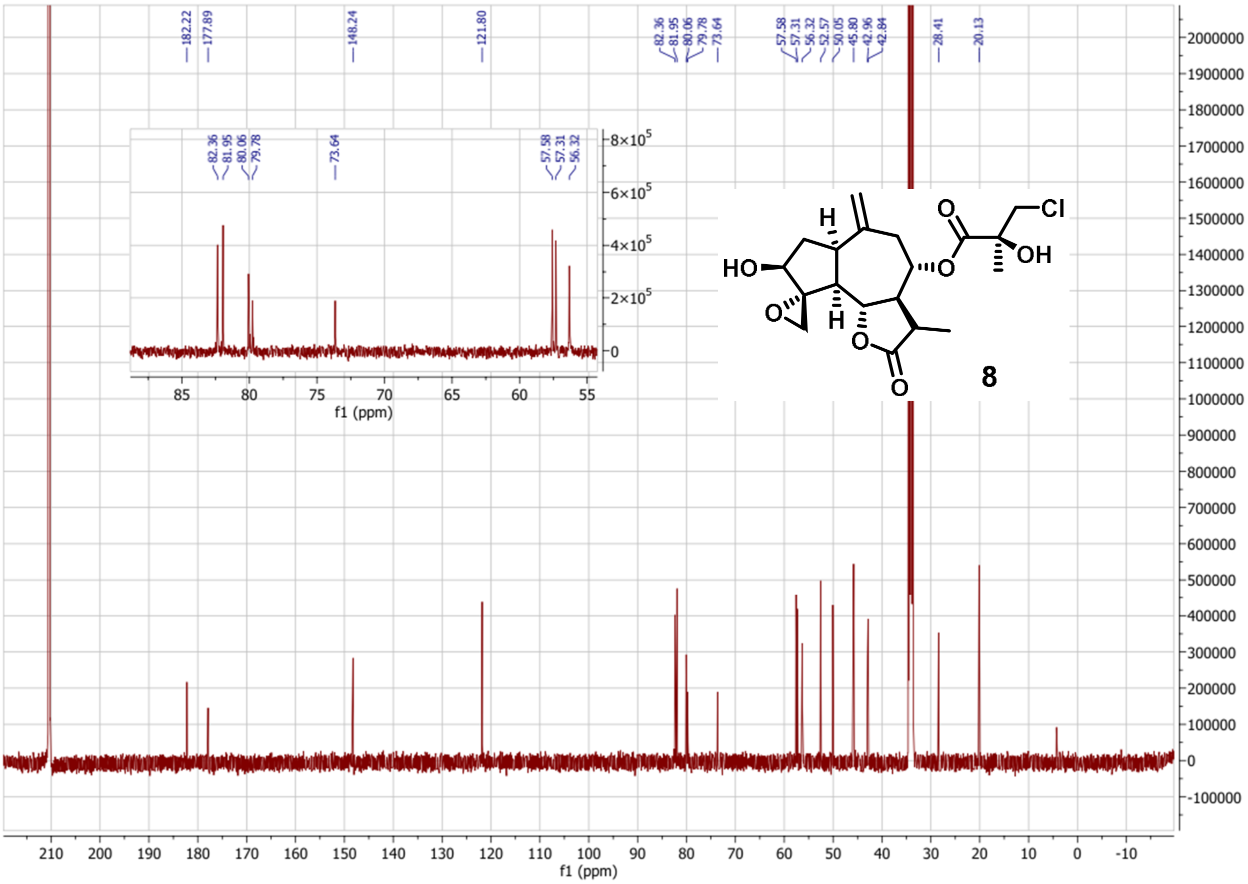

Figure S31: ^{13}C-NMR (125 MHz, Acetone-d6) Spectrum of Compound **8**

Elemental Composition Report

Page 1

Multiple Mass Analysis: 2 mass(es) processed
Tolerance = 5.0 PPM / DBE: min = -3.0, max = 120.0
Element prediction: Off
Number of isotope peaks used for i-FIT = 3

Monoisotopic Mass, Even Electron Ions
117 formula(e) evaluated with 2 results within limits (all results (up to 1000) for each mass)
Elements Used:
C: 0-20 H: 0-25 O: 0-7 Na: 0-1 35Cl: 0-1 37Cl: 0-1

Ignacio B
(ESI-20-683) Ignacio B (IB -CL-24) 104 (4.490)

1: TOF MS ES+
2.78e+003

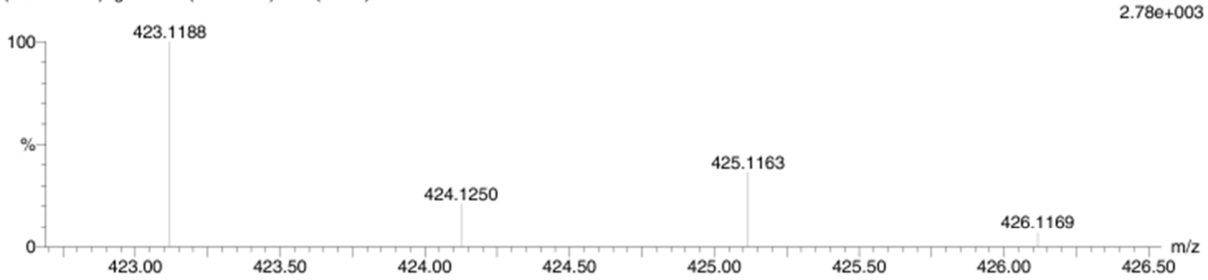

Minimum:	23.00				-3.0			
Maximum:	100.00		5.0	5.0	120.0			
Mass	RA	Calc. Mass	mDa	PPM	DBE	i-FIT	i-FIT (Norm)	Formula
423.1188	100.00	423.1187	0.1	0.2	6.5	24.9	0.0	C19 H25 O7 Na 35Cl
425.1163	36.40	425.1157	0.6	1.4	6.5	20.2	0.0	C19 H25 O7 Na 37Cl

Figure S32: HRESI-MS Spectrum of Compound **8**

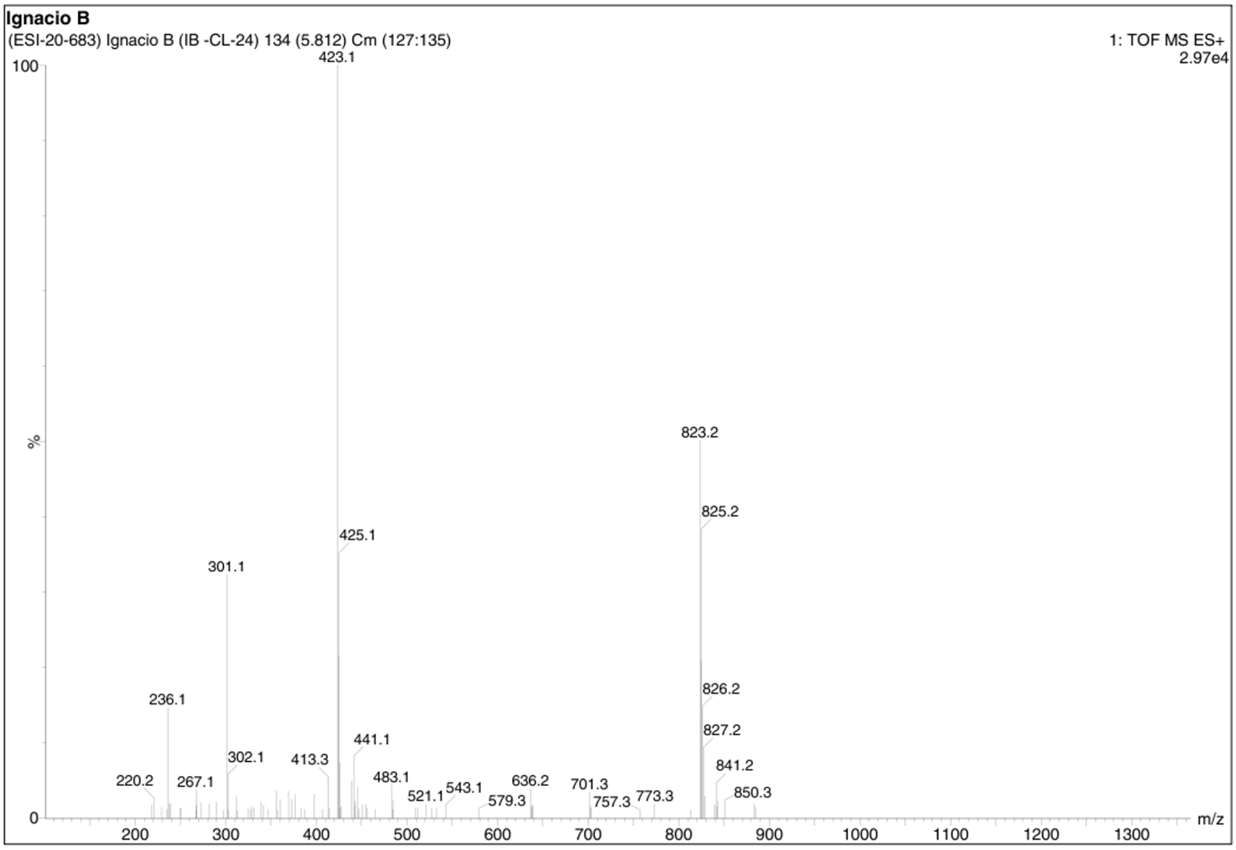

Figure S33: ESI-MS Spectrum of Compound **8**
